# Universal and transferable attacks on pathology foundation models using microscopic perturbations

**DOI:** 10.1038/s41377-026-02347-w

**Published:** 2026-06-01

**Authors:** Yuntian Wang, Xilin Yang, Che-Yung Shen, Shuhang Dong, Nir Pillar, Aydogan Ozcan

**Affiliations:** 1 Electrical and Computer Engineering Department, University of California, Los Angeles, CA USA; 2 Bioengineering Department, University of California, Los Angeles, CA USA; 3 California NanoSystems Institute (CNSI), University of California, Los Angeles, CA USA; 4 Department of Mathematics, University of California, Los Angeles, CA USA; 5 Department of Pathology, Hadassah Hebrew University Medical Center, Jerusalem, Israel; 6 Department of Surgery, University of California, Los Angeles, CA USA

**Keywords:** Microscopy, Biophotonics

## Abstract

The advent of foundation models initiated a paradigm shift in pathology and optical microscopy. However, these powerful systems also introduce vulnerabilities, making them susceptible to adversarial attacks. To shed light on these potential threats, here we introduce Universal and Transferable Adversarial Perturbations (UTAP) for pathology foundation models that reveal critical vulnerabilities. Optimized using deep learning, UTAP comprises a fixed and weak microscopic noise pattern that, when added to a pathology image, systematically disrupts the feature representation capabilities of foundation models. Therefore, UTAP induces performance drops in downstream tasks that utilize foundation models, including misclassification across a wide range of unseen data distributions. We demonstrate two key features of UTAP: (1) *universality*: its microscopic perturbation can be applied across diverse field-of-views independent of the dataset that UTAP was developed on, and (2) *transferability:* its perturbation can successfully degrade the performance of various external, black-box pathology foundation models—never seen before. These indicate that UTAP is not a dedicated attack associated with a specific foundation model or microscopy image dataset, but rather constitutes a broad threat to pathology foundation models and their applications. We evaluated UTAP across various state-of-the-art pathology foundation models on multiple datasets, causing significant drops in their performance with visually imperceptible microscopic modifications to the input images using a fixed noise pattern. The development of these potent attacks establishes a benchmark for model robustness evaluation, highlighting a need for advancing defense mechanisms to ensure the safe/reliable deployment of AI in pathology and optical microscopy.

## Abstract (web summary)

By applying a single, optimized microscopic noise pattern, universal and transferable attacks expose vulnerabilities in pathology foundation models.

## Graphical abstract

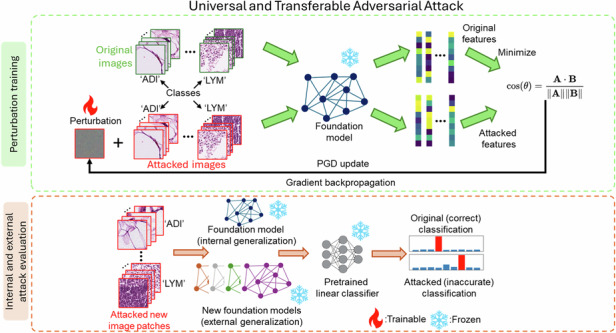

## Introduction

The integration of Artificial Intelligence (AI) into computational pathology, utilizing general-purpose foundation models, has significantly enhanced performance on a vast range of tasks, including cancer detection, subtyping, grading, and staging^1–6^, compared to task-expert AI models that specialize in only one task. Such foundation models are predominantly self-supervised by either contrastive learning or masked modeling^7–11^ and effectively utilize Vision Transformers (ViT)^12^ as their backbone. These foundation models are pre-trained at large scale using, e.g., millions of pathology slides, and then used as feature extractors for diverse downstream tasks^13–17^. The self-attention mechanism—central to ViTs—enables long-range context, but its global token coupling can also be exploited for attacks: attention-aware or patch-based adversarial methods have shown effective targeted perturbations against ViTs^18–25^. These adversarial attacks, utilizing subtle and carefully crafted perturbations, are often imperceptible to human observers but can induce catastrophic model failures^25–27^, ranging from representation power collapse to attacker-directed hallucinations. Such an attack could, for instance, cause a model to misdiagnose a malignant tumor as benign, posing a direct and severe risk to patients, especially without human experts in the loop.

In the domain of adversarial attacks, earlier efforts predominantly focused on a single or a small set of specific image samples or models, resulting in attacks that are highly specialized in scope^18,21^. In this work, we move beyond model- or image patch-specific attacks to explore more broadly applicable threats to pathology foundation models characterized by both *universality* and *transferability*. For exploring the vulnerabilities of pathology-related foundation models, we developed a universal and transferable adversarial perturbation (UTAP) framework, which utilizes an adaptive Projected Gradient Descent (PGD)-based^28^ optimization process to iteratively craft a subtle microscopic noise pattern. We define universality as the capability of a single, fixed microscopic perturbation that applies across a wide range of models and data, including unseen images from different datasets, to cause model failure. We define transferability as an attack crafted against one foundation model, where it can successfully compromise, without any adjustments to its microscopic perturbation, other foundation models, which are never seen before, i.e., neither the parameters nor the gradients of the attacked models are accessible to the attacker. We improved the universality and transferability of our adversarial attacks with two key designs. First, we iteratively optimize the attack across a set of training data to disrupt the model’s output feature space by minimizing the similarity between the feature representations of the original and perturbed/attacked microscopic images. This strategy fundamentally hampers the model’s representational power and grants the perturbations superior universality across various image patches. Second, we add random masking and attention dropping regularization when computing the gradients to ensure better transferability to other foundation models never seen before, i.e., following a black-box setting. The efficacy of UTAP was comprehensively validated by demonstrating that a single, fixed microscopic perturbation successfully degrades the performance of seven state-of-the-art pathology models^29–35^, significantly reducing the probing accuracy in downstream classification tasks while simultaneously compromising the representation power of all the tested models.

UTAP shows that one universal microscopic perturbation trained in one setting transfers to unseen datasets and other foundation models without any modifications, revealing system-level vulnerabilities that standard evaluation methods can miss. To support safer development and deployment of pathology foundation models, it is essential to comprehensively study these threats and develop robust defenses. Framed as “ethical hacking”, our work develops and benchmarks advanced attacks and aims to raise awareness, with the goal of enabling powerful tools needed to build, assess, and improve the next generation of resilient, secure computational pathology and optical microscopy models.

## Results

### Universal and transferable adversarial attacks on pathology foundation models

**Fig. 1:**
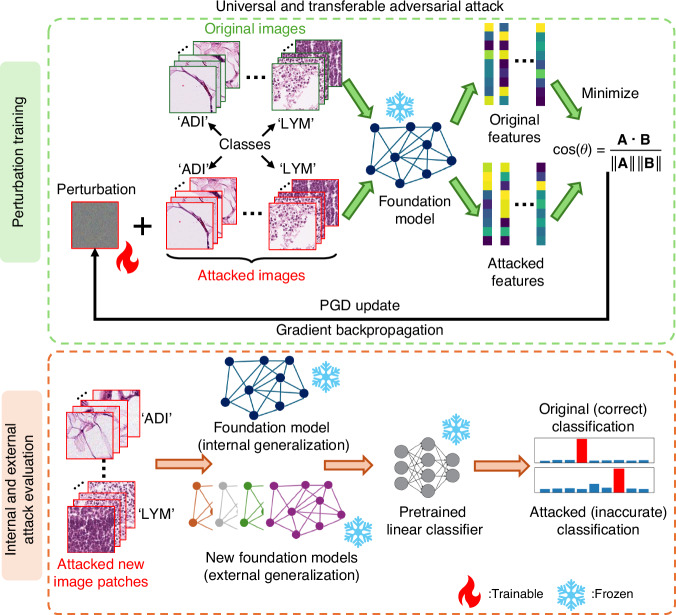
Workflow of universal and transferable adversarial perturbation (UTAP) training and evaluation. Top row shows the process of UTAP training which minimizes the cosine similarity of original and attacked microscopic features extracted from a frozen foundation model. The bottom row is the evaluation process of UTAP attack, where the attacked images were passed through a frozen foundation model used for training (internal generalization) and unseen, black-box foundation models (external generalization) cascaded with their respectively pretrained linear classifiers. The fire and snowflake symbols indicate whether the parameters are trainable or frozen/fixed

We first validate our attack strategy on a downstream classification task using a dataset of 100,000 non-overlapping patches from hematoxylin and eosin (H&E) stained histological images of human colorectal cancer (CRC) and normal tissue^36^. As illustrated in Fig. 1 and Algorithm 1 in the “Methods” section, the attack generation scheme involves iteratively optimizing a single, trainable UTAP microscopic pattern via the adaptive PGD^28^ method with random masking and attention dropping regularization^37^. For details of the implementation, refer to the Methods section. During the training, the UTAP-generated noise pattern is added to original microscopy images, and both the original and the attacked images are passed through a frozen pathology foundation model to extract their respective feature representations. The core of our optimization aim is to minimize the cosine similarity between the original and attacked feature embeddings, thereby forcing them to be as dissimilar as possible in the feature space, corrupting the representation power of the foundation model. Once the training converged and the perturbation is finalized, the UTAP’s effectiveness was comprehensively evaluated using a methodology to assess universality and transferability concurrently. To assess the performance of classification downstream tasks, we first trained a light-weight linear classifier on the extracted features from clean images (classification tokens / CLS tokens) in the classification training dataset and then computed the blind testing classification accuracy on the corresponding test dataset; this sets the baseline accuracy in each task before the attack (see Methods for details). We then applied a single, fixed UTAP perturbation pattern to the images in the same testing dataset that neither the classifier nor UTAP saw during their optimization. These perturbed/attacked microscopy images were then evaluated against multiple foundation models, including the original model used during UTAP optimization (internal generalization) and new, unseen foundation models (external generalization). The attack’s success was quantified by the drop in the classification accuracy in each case—computed as the difference between the original and attacked accuracy values—using the well optimized pre-trained linear classifier for each foundation model and data combination. This testing pipeline allows us to confirm the attack’s universality by quantifying its impact on new, unseen data, and its transferability by assessing the performance degradation across different foundation models – never seen before.

**Fig. 2:**
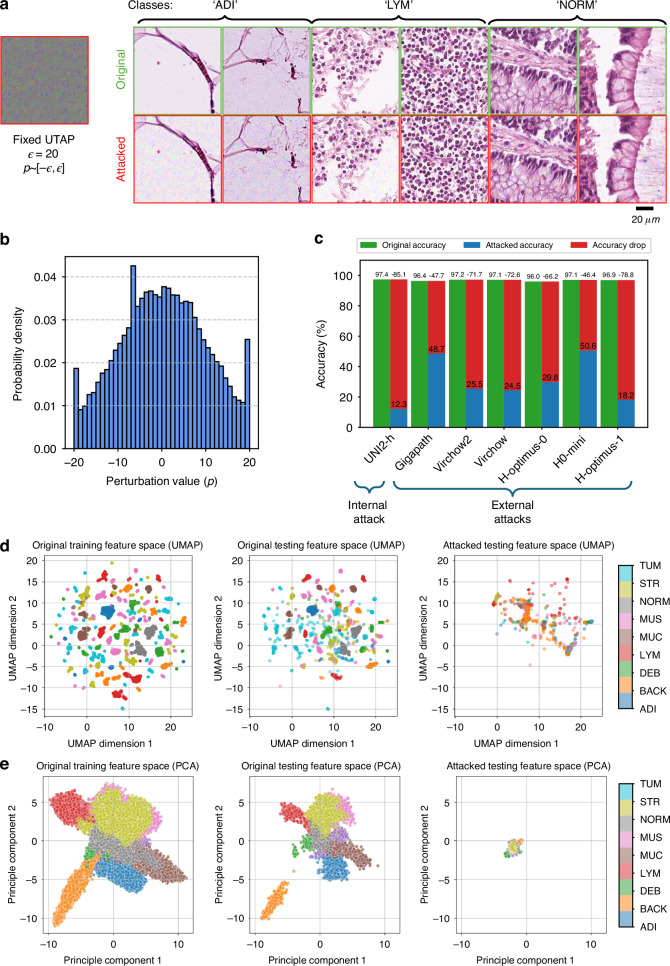
UTAP attack results on CRC-100K dataset. **a** Visualization of the optimized UTAP with the original and attacked microscopic images of three classes (‘ADI’ for adipose, ‘LYM’ for lymphocytes and ‘NORM’ for normal colon mucosa) sampled from the CRC-100K dataset. **b** The histogram of UTAP perturbation, bounded in the range of $\pm \epsilon$, where $\epsilon =20$. **c** The original (green), attacked (blue) and dropped (red) classification accuracy values on the internal attack (the foundation model used for UTAP training) and various external attacks (for unseen foundation models). **d** the UMAP projections into the 2D space of the original features and the attacked microscopic features. **e** The PCA projections into the 2D space of the original features and the attacked microscopic features

The efficacy of the UTAP attack was qualitatively and quantitatively evaluated in Fig. 2. The perturbation pattern itself, visualized against a neutral gray background in Fig. 2a, is composed of pixel values strictly bounded within a small range of $[-\epsilon ,\epsilon ]$. As a direct consequence of this constraint, when the UTAP perturbation is added to the original images, the resulting attacked images are visually almost indistinguishable from their unperturbed counterparts. This perceptual subtlety is also demonstrated in Fig. 2a with a side-by-side comparison of the original and attacked tissue patches from three distinct classes: adipose (ADI), lymphocyte (LYM), and normal colon mucosa (NORM). Further analysis of UTAP’s statistical properties is reported in Fig. 2b, which displays a histogram of its pixel-wise values averaged from the three-color channels. The histogram plot reveals a distribution centered approximately at zero and exhibits prominent spikes at the $\pm \epsilon$ bounds, a characteristic outcome of the hard energy constraints enforced by the adaptive PGD optimization algorithm used to generate the attack (see the Methods section).

While UTAP is designed to be visually imperceptible, its impact on model performance is substantial. The impact of the attack on classification accuracy was quantitatively reported in Fig. 2c, which details the attack’s performance across seven different foundation models while we only used one foundation model (UNI2-h) for training UTAP. The chart compares the original classification accuracy of each model (green bars) with the accuracy on the images attacked by UTAP (blue bars). The substantial drop in performance, quantified for each model (red bars), demonstrates the attack’s ability to induce significant model failure. Critically, this degradation is observed not only in the model used to train the perturbation (UNI2-h in this case) but across all the other six external, black-box foundation models (the weights and gradients of which were *not* known), confirming the attack’s high degree of transferability. It is important to note that this accuracy drop is not caused by an attack on the downstream linear probing layer, but rather stems directly from the corruption of the upstream feature representations, which is also confirmed with the analysis in Fig. 2d-e. To shed more light on our attack’s impact on the feature space representation, Fig. 2d displays the Uniform Manifold Approximation Projections (UMAP)^38^ of the feature embedding of image patches extracted by the foundation model before and after the attack. The left and middle scatter plots show that in the absence of an attack, the training and testing feature embeddings of different tissue classes form distinct, well-separated clusters through UMAP. In contrast, the right scatter plot reveals that after applying the optimized UTAP perturbation to the test images, these orderly clusters collapse into chaotic and largely undifferentiated structures. This dimensionality reduction confirms that the UTAP operates by fundamentally corrupting the foundation model’s feature representation capability, causing the feature space to collapse, destroying the linear separability of features, and thereby crippling its discriminative classification performance. Figure 2e further illustrates this effect through the Principal Component Analysis (PCA) of the feature representations in addition to UMAP. These visualization results confirm a similar insight that the attacked features undergo a mode collapse, forming a tight, squeezed cluster in contrast to the well-separated original features. These analyses demonstrated that UTAP was able to not only cause significant classification accuracy drops, but also compromise the general representation power of the foundation models, even for black-box foundation models that were never seen before.

To further understand the effect of the UTAP attack and its behavior, we compared the probabilities predicted by the linear classifier from the original image data versus the probabilities resulting from the attacked images. Supplementary Fig. S1a presents the results of the attack on the UNI2-h model alongside the corresponding original and attacked images shown on the left for all nine data classes. The results highlight a key difference between UTAP and traditional adversarial attack methods. Conventional attacks are typically targeted, i.e., they have a predefined attack goal, manipulating inputs to maximize the probability for a specific incorrect class^28,39^. In contrast, as shown in Supplementary Fig. S1b, UTAP focuses on degrading the feature space itself, without a fixed directional preference, and therefore it does not manifest itself as a consistent peak for any single incorrect class, but rather generally corrupts the model’s confidence, resulting in a more uniform distribution across all false classes. This non-targeted behavior is further demonstrated in Supplementary Fig. S2, which compares the classification probability distributions for the internal foundation model (UNI2-h) against several external foundation models (e.g., Gigapath and Virchow2), which were not used during the training phase. The figure reveals that while the attack degrades performance across all the foundation models, the specific characteristics of the resulting probability distributions are model-dependent. These variations on the predicted probability distributions underscore that UTAP does not force a single, predictable outcome. Instead, it interacts with each foundation model with a unique behavior, providing further evidence that it operates by fundamentally disrupting the feature representation capabilities with high transferability to black-box foundation models, never seen before. Interestingly, the UTAP attack exhibits relatively lower efficacy on smooth muscle (MUS) samples across the evaluated models. This partial resilience can be attributed to the inherent morphological properties of smooth muscle, which is substantially more homogenous compared to other tissue types, presenting a dense, relatively uniform eosinophilic texture. Compared to other classes, such as adipose tissue, which is dominated by empty background space, or tumor and lymphocyte regions characterized by high textural variation, smooth muscle presents consistent, low-frequency structural features. Consequently, the high-frequency microscopic noise introduced by UTAP presents limitations to fully disrupt these uniform representations.

**Fig. 3:**
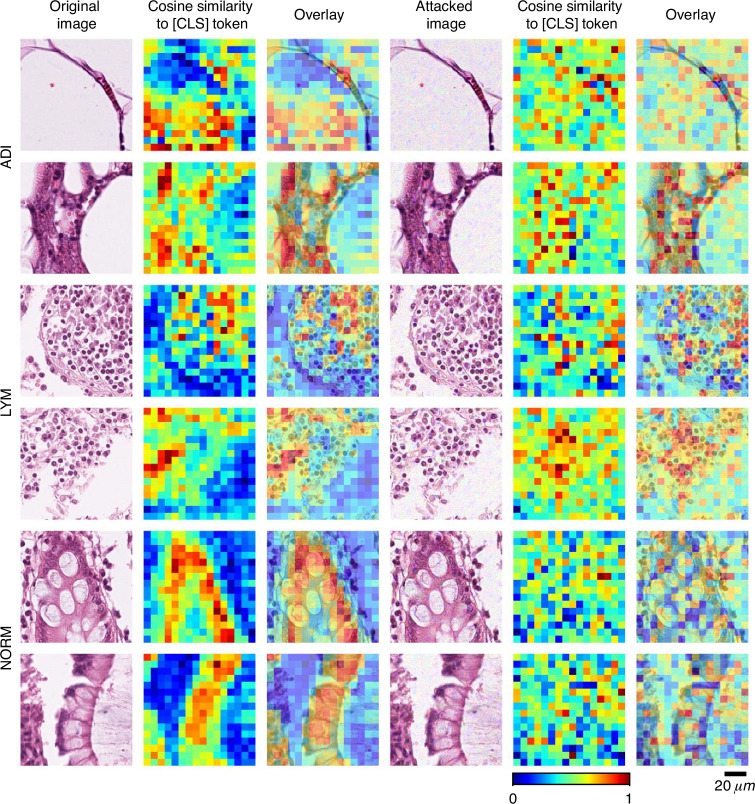
Original and UTAP attacked attention maps extracted by a pathology foundation model (UNI2-h). Visualization of the cosine similarity between the [CLS] token and all positional patch tokens for tissue samples from three different classes (ADI, LYM, and NORM). For the original images (left column), the foundation model exhibits coherent attention, with high similarity (red color) correctly localized on salient tissue structures, as desired and expected. After applying the UTAP microscopic attack (right column), these attention patterns result in disorganized and scattered states. This provides direct evidence that UTAP fundamentally corrupts the foundation model’s core microscopic feature extraction capabilities. The attention maps are calculated using UNI2-h model

We further investigated UTAP attack’s impact on the model’s spatial features by visualizing the cosine similarity between the classification/[CLS] tokens and all the local visual tokens (see the Methods) and plotting them as a heatmap in the corresponding locations on the whole slide image. As illustrated in Fig. 3, before the attack, the resulting heatmaps clearly show the foundation model’s focus on diagnostically relevant tissue structures, indicated by high similarity (red hues), while assigning low similarity values to irrelevant regions (blue hues). Following the application of UTAP perturbation, the attacked attention maps become profoundly disrupted, exhibiting a scattered and incoherent distribution of cosine similarity values. This degradation provides direct evidence that our attack does not merely manipulate a global decision boundary but fundamentally impairs the model’s core ability to extract meaningful and localized features across the entire image, thereby compromising its foundational understanding and representation of the tissue content.

To further assess the universality of UTAP across different data distributions, we also evaluated the attack’s impact on an entirely unseen dataset comprising six selected classes from The Cancer Genome Atlas Program (TCGA) Uniform Tumor dataset^40^, none of which were used during the optimization process. As illustrated in Supplementary Fig. S3, the application of the pre-optimized UTAP microscopic pattern, without any adaptation, induced clear visual perturbations alongside substantial performance degradation, resulting in classification accuracy drops ranging from 16.1% to 51.6% across the evaluated foundation models. To rigorously validate this universality of UTAP, we subsequently increased the number of out-of-distribution (OOD) datasets tested. We assembled a comprehensive external test suite that additionally included BACH^41^, BreakHis^42^, GCHTID^43^, and LC25000^44^ datasets. The pre-optimized UTAP pattern continued to induce severe performance degradations across all models on these newly introduced datasets (Supplementary Fig. S4). For instance, the attack caused the classification accuracies to plummet to as low as ~22% on LC25000 and ~14% on GCHTID. This successful and consistent attack on a diverse array of OOD images further demonstrates the universality of the UTAP microscopic perturbation. It indicates that UTAP successfully exploits dataset-agnostic, feature-level vulnerabilities, thereby underscoring a broader security risk that extends well beyond the source distribution.

To validate the general applicability and cross-architecture transferability of the UTAP framework beyond ViT architectures, we also extended our external generalization evaluation to include Convolutional Neural Network (CNN)-based models. Specifically, we applied the fixed UTAP microscopic perturbation—originally optimized on a ViT backbone—directly to the input images processed by two CNN-based pathology models, i.e., KimiaNet^45^ and CSCO^46^. We evaluated the attack’s efficacy across a diverse array of datasets, including the original test distribution. As illustrated in Supplementary Fig. S5, the UTAP microscopic perturbation successfully induced significant performance degradation across both CNN models and all evaluated datasets, without any architecture-specific adaptation or retraining. For example, on the LC25000 dataset, the classification accuracy dropped from 92.75% to 46.89% for KimiaNet, and from 83.14% to 20.01% for CSCO. Similarly, substantial accuracy drops were observed on the TCGA-TU dataset, decreasing from 87.21% to 57.51% for KimiaNet and from 51.03% to 11.59% for CSCO. These results demonstrate that the fundamental feature representation collapse induced by UTAP is not strictly bound to the spatial tokenization mechanism of ViTs. Instead, the microscopic perturbation of UTAP acts as a highly transferable, universal feature distractor capable of compromising the global feature aggregation of entirely different neural network architectures.

**Fig. 4:**
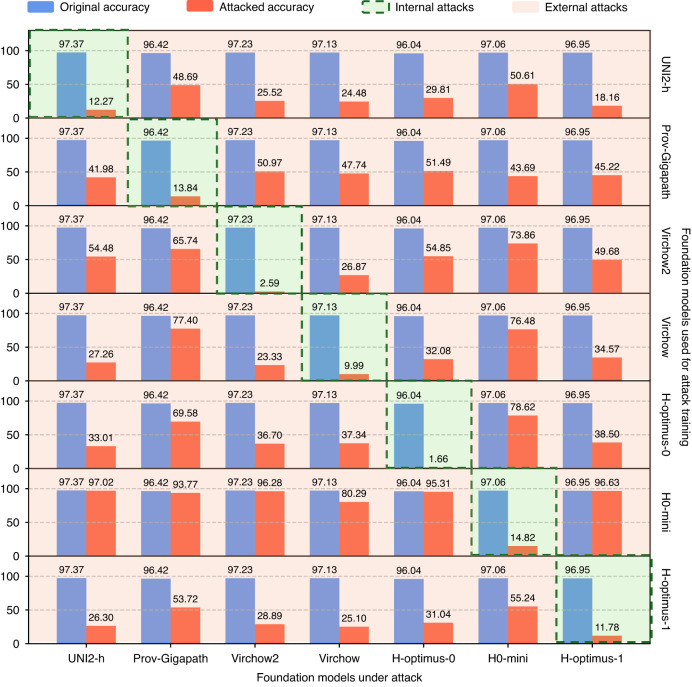
Cross-model transferability of UTAP. The matrix displays the attack performance of UTAP perturbations trained on each foundation model (rows) when evaluated against all the other foundation models in the test set (columns). The diagonal elements, highlighted by the green dashed boxes, represent the internal attacks, while the off-diagonal elements represent the external attacks on new foundation models never seen before. In each subplot, the blue bar indicates the original accuracy of the target model, and the red bar shows the accuracy after the UTAP attack

To further quantify the transferability of UTAP, we conducted a systematic cross-model attack analysis, summarized in Fig. 4. UTAP optimization in this analysis used the same intensity constraint and step schedule, and evaluation is performed on unseen patches for every model; see the Methods for details. This evaluation is structured as a matrix-like representation where the row indexes represent the foundation model used to train the UTAP microscopic perturbation that is used, while the column indexes represent the foundation model being attacked and evaluated. The diagonal entries, highlighted by the dashed green boxes, correspond to internal attacks, where the perturbation is evaluated on the same foundation model architecture it was developed on. The off-diagonal elements represent external attacks, simulating a black-box scenario where a perturbation trained on one foundation model is directly used to attack other unseen foundation models. Crucially, all attacks presented in this analysis were performed on new, unseen image patches. The results of this analysis reveal two critical findings. First, the internal attacks show that the UTAP perturbation process is consistently powerful, reducing accuracy on the models on which it was trained. More importantly, the external attack results reveal that UTAP exhibits a high degree of transferability across all training-testing model pairs. For instance, a UTAP perturbation trained on UNI2-h not only reduces its own accuracy down to 12.27% but also successfully degrades the performance of other foundation models, such as lowering Prov-Gigapath’s accuracy from 96.42 to 48.69% and Virchow2’s accuracy from 97.23% to 25.52%.

We further evaluated the transferability of UTAP by training it with multi-source optimization, in which the source model is randomly switched every eight steps from a fix-sized pool drawn from seven foundation models (detailed in Supplementary Fig. S6). All UTAP optimization steps used the same intensity constraint and step schedule, and evaluation is performed on unseen image patches for every model. In each optimization process, the foundation models included in the pool are treated as internal (dashed green box), while the excluded models are treated as external (orange box). The results reveal two key trends. First, the foundation models present in the training pool experience markedly larger accuracy drops than external models, indicating that direct exposure to the foundation model during UTAP training still confers an advantage to the attack. Second, as the pool size increases, transfer to external foundation models becomes broader and more uniform in their attack performance, but the per-model impact on internal targets relatively diminishes. Intuitively, optimizing the attack against a diverse group of foundation models steers the perturbation toward features shared across these models (which improves transfer success), while conflicting gradients prevent over-specialization to any single model weight or architecture (reducing peak severity). Practically, this suggests that an attacker can tune the size of the model pool to balance maximal depth on a specific model versus wide, cross-model disruption. With these analyses, we demonstrated the ability of UTAP to significantly degrade the performances of multiple, unseen external foundation models; these results underscore a critical vulnerability, highlighting a need for advanced defense mechanisms that are robust against not only direct attacks but also these more insidious, transferable threats.

**Fig. 5:**
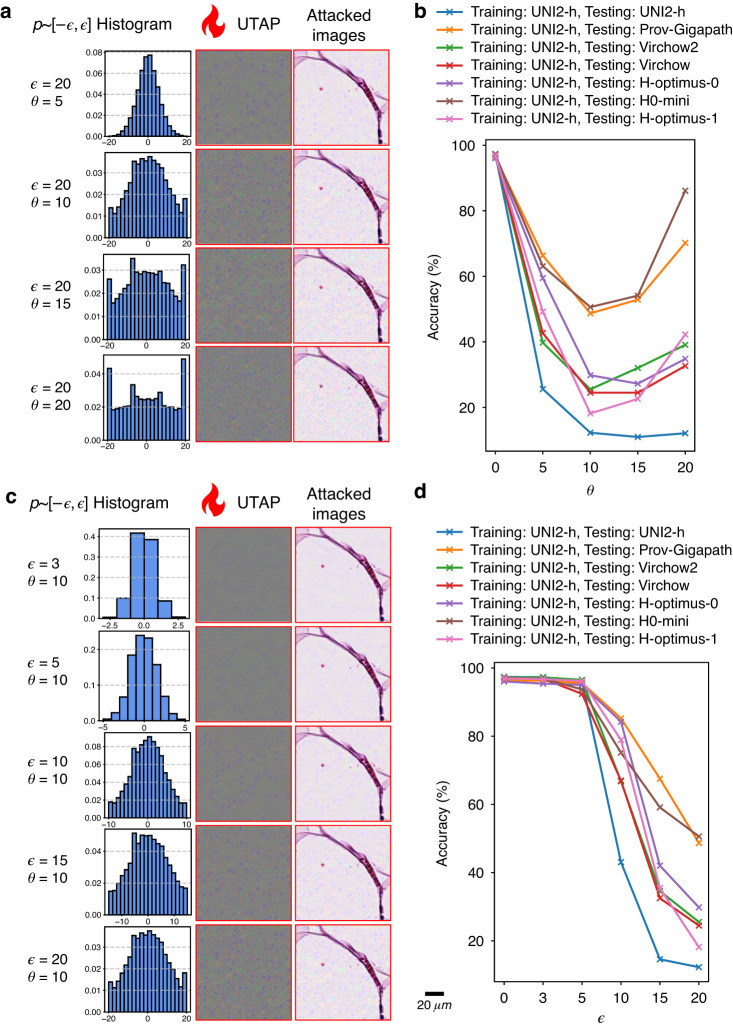
Ablation study on UTAP hyperparameters *θ* and *ϵ.* **a** Visualization of UTAP attack with a fixed perturbation magnitude $\epsilon$ and varying step-size scaling factor $\theta$. Each row shows the resulting perturbation histogram, UTAP visualization, and the attacked microscopic image. **b** The classification accuracy of seven foundation models (including internal and external attacks) as a function of $\theta$. **c** Visualization of UTAP attacks with a fixed $\theta$ and varying $\epsilon$. **d** The classification accuracy of seven foundation models (including internal and external attacks) as a function of $\epsilon$

Having established how training diversity modulates transferability, next we examine how optimization choices shape UTAP’s performance by exploring two key hyperparameters: $\theta$ which is the scaling factor for the step size and $\epsilon$ which is the allowable magnitude of the perturbation; see Fig. 5. We first analyzed the impact of $\theta$, by training all UTAP perturbations on the UNI2-h model and evaluating them across all seven foundation models while varying $\theta$ ($\epsilon$ fixed). The histogram of the UTAP perturbations and the attacked images for different choices of $\theta$ are visualized in Fig. 5a. In these results, we observe that larger $\theta$ drives the perturbation distribution to become more bimodal, concentrating at the $\pm \epsilon$ boundaries. The quantitative accuracy evaluations reported in Fig. 5b reveal a distinct difference between the internal and external attack performances. The internal attack efficacy on UNI2-h increases monotonically with $\theta$, whereas the external attack efficacy is non-monotonic: the highest accuracy drop is observed at an intermediate $\theta =10$ and then weakens. Thus, step size critically governs transferability, with an optimal $\theta$ value balancing internal strength and cross-model generalization of the attack. Similarly, with $\theta$ being fixed, we also analyzed the impact of $\epsilon$—the perturbation bound—which sets the maximum magnitude of the perturbation pattern. The analyses and visualizations reported in Fig. 5c reveal that as $\epsilon$ increases, the perturbation becomes stronger and as anticipated, the attack gets visually noticeable on the resulting images. This increase in perturbation strength correlates directly with attack efficacy, as shown in Fig. 5d: accuracy drops due to the attack grow monotonically with increasing $\epsilon$ across all the targeted models. Consequently, $\epsilon$ governs the potency–stealth trade-off: larger bounds yield stronger but more perceptible attacks.

To better understand the specific advantages of our proposed methodology, we expanded the ablation study to evaluate the optimization objective and the individual regularization components used in UTAP training. First, we compared the proposed cosine-similarity objective against a standard cross-entropy loss. The results reported in Supplementary Fig. S7a reveal that utilizing a cross-entropy objective significantly reduces the attack’s overall efficacy, yielding noticeably diminished transferability across all external foundation models compared to the cosine-similarity objective. This confirms that directly decorrelating feature embeddings via cosine similarity is a substantially more potent strategy for driving representation collapse than logit-level manipulation. Second, we evaluated the efficacy of UTAP trained with and without the combined and individual regularization techniques (attention dropping and random masking). The results reveal an intuitive trade-off: removing all regularization techniques boosts the attack’s efficacy against the training foundation model (resulting in improved *internal* attacks) but significantly compromises its transferability to external models. For example, omitting only random masking led to the most severe internal overfitting and the poorest external transferability. Similarly, omitting only the attention-dropping regularization yielded suboptimal transferability compared to the combined approach. This is because unregularized or partially regularized perturbations overfit to the training model by exploiting model-specific attention patterns. As these patterns are not strictly conserved across different foundation models, the perturbation fails to generalize, diminishing its effectiveness in black-box attacks to other foundation models that have never seen before.

Furthermore, to benchmark the overall potency of the UTAP framework, we compared its performance against another adversarial baseline using the Fast Gradient Sign Method (FGSM)^39^. As illustrated in the comparative analysis in Supplementary Fig. S7b, UTAP consistently and significantly outperformed FGSM across all evaluated architectures. While FGSM induced moderate performance degradation—for instance, reducing the internal UNI2-h model accuracy to 67.2% and the external Virchow2 accuracy to 70.5%—UTAP achieved profoundly more severe classification drops to 12.3% and 25.5%, respectively. This distinct performance gap, observed uniformly across the entire external model suite, confirms that UTAP methodology is substantially more effective at generating highly transferable, universal adversarial perturbations than traditional gradient-based techniques.

### Patch-specific and class-specific adversarial perturbations

**Fig. 6:**
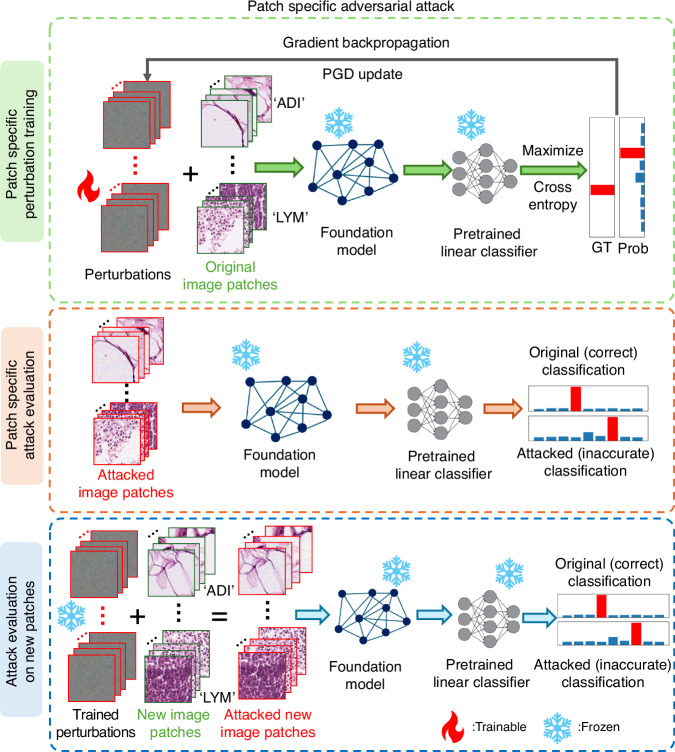
Workflow of Patch Specific Adversarial Perturbation (PSAP) training and evaluation. The top row shows the process of PSAP training, where the patch-specific microscopic perturbations were added to the original image patches and were optimized to maximize the cross entropy of the ground truth label and the classification probability extracted from the pretrained frozen linear classifier. The remaining rows show the process of PSAP evaluation on trained (middle row) and new (bottom row) image patches. The fire and snowflake symbols indicate whether the parameters are trainable or frozen/fixed

**Fig. 7:**
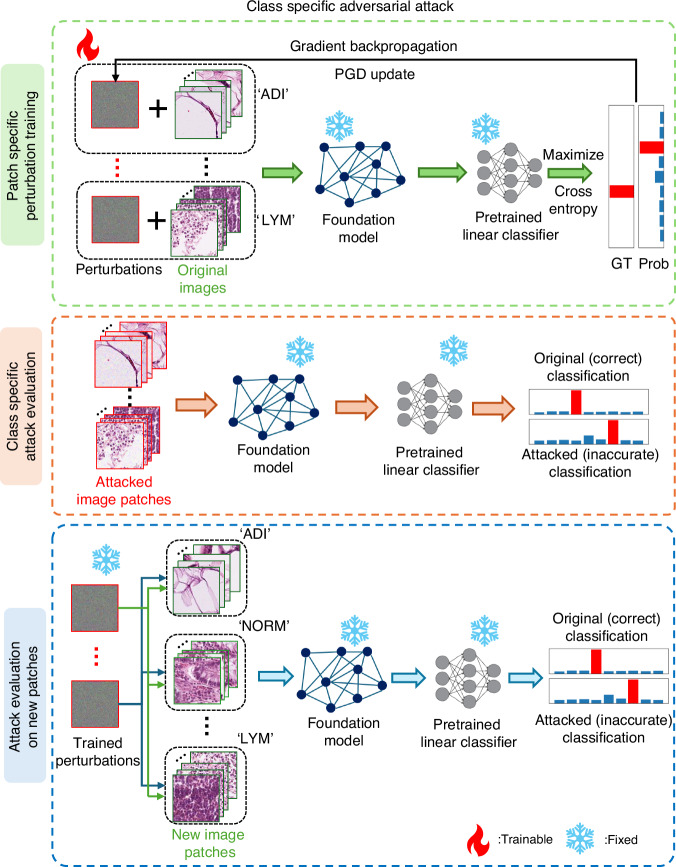
Workflow of Class Specific Adversarial Perturbation (CSAP) training and evaluation. The top row shows the process of CSAP training, where the class-specific microscopic perturbations were added to the original image patches of a specific data class and were optimized to maximize the cross entropy of the ground truth label and the classification probability extracted from the pretrained frozen linear classifier. The remaining rows show the process of CSAP evaluation on trained (middle row) and new (bottom row) image patches. The fire and snowflake symbols indicate whether the parameters are trainable or frozen/fixed

Next, we consider image patch-specific and data class-specific adversarial perturbations; see Figs. 6–7. Consider a scenario in which an adversary targets a specific patient whose tissue specimen is submitted for diagnostic analysis. Depending on the adversary’s prior knowledge of the patient, the required universality of the attack varies along a hierarchy from the most to the least specific. At the most specific level (low universality), if the attacker obtains an exact digital copy of the patient’s tissue patch, they can craft a bespoke perturbation; we term this attack as **P**atch-**S**pecific **A**dversarial **P**erturbation (PSAP), which optimizes a unique perturbation pattern per image patch for an adversarial attack. With only class-level knowledge (e.g., the specimen’s diagnostic category), the attacker can also deploy a class-conditional perturbation; we model this scenario using the **C**lass-**S**pecific **A**dversarial **P**erturbation (CSAP) to cause misclassification of a specific data class. At the least specific/most universal strategy, an attacker possesses no prior knowledge of the specific image or its class label, necessitating a universal attack (UTAP) that can degrade model performance across all inputs and models – as we reported in our former sub-section. At the lowest level of universality, we evaluate image patch-specific adversarial perturbations (shown in Fig. 6), where an attacker has knowledge of a targeted, specific sample image. In this attack strategy, each image patch receives a trainable perturbation, which is added to the corresponding patch before the attacked image is processed by the frozen pathology foundation model and its pre-trained linear classifier. To induce misclassifications, the perturbations are iteratively optimized by maximizing the cross-entropy loss between the model’s prediction and the one-hot ground-truth label (detailed in the Methods section). This attack strategy reveals a critical trade-off between internal attack performance and external generalization: tailored to individual image patches, PSAP collapses the classification accuracy on the optimized samples from 97.37% to 0.00% where none of the predictions are correct. However, the attack fails in terms of universality, leaving performance on unseen images largely intact (95.00% classification accuracy). Thus, while patch-specific attacks can severely compromise a model on specific image data, their generalization capability is limited since the attack design is patch-specific.

**Fig. 8:**
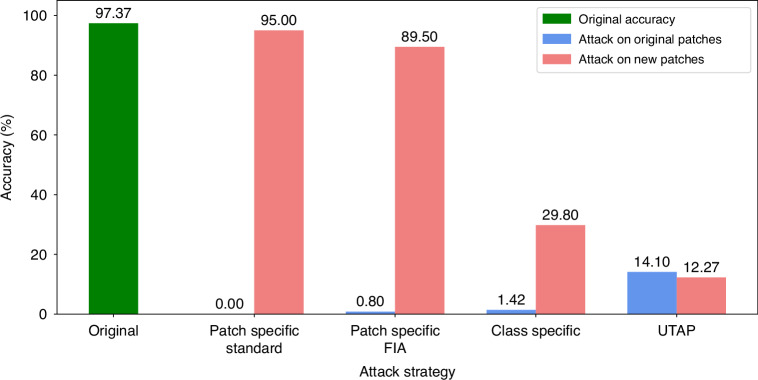
Comparison of adversarial attack strategies and their generalization. The green bar indicates the original classification accuracy. The classification accuracies were evaluated on CRC-100K dataset using the UNI2-h foundation model. The attacked classification accuracy values on the original/training and new/testing image patches are shown with the blue and red bars, respectively. The methods of applying these three categories of microscopic perturbations are shown in Figs. 1, 6 and 7

To further benchmark the behavior of patch-specific attacks against other adversarial techniques, we also evaluated the Feature Importance-aware (FIA) attack method. Recognizing FIA’s objective to disrupt critical, object-aware features, we implemented it as a patch-specific adversarial perturbation within our evaluation framework^47^. Consistent with the severe overfitting observed in the standard PSAP optimization, the FIA attack successfully compromised the model on the targeted instances, reducing the classification accuracy to 0.8% on the original image patches. However, it demonstrated limited generalization capabilities; when these FIA-generated perturbations were applied to new, unseen image patches, the classification accuracy remained robust at 89.5% (see Fig. 8). This comparative analysis reported in Fig. 8 further substantiates the observation that while advanced patch-specific gradient methods can catastrophically subvert model predictions on isolated samples, their highly tailored optimization landscape fundamentally restricts their universality and transferability across broader data distributions.

Next, we evaluated class-specific adversarial perturbations that target all images within a single tissue class (see Fig. 7). A single perturbation was trained per class—for example, one fixed perturbation pattern for all adipose (ADI) images and another one for all lymphocyte (LYM) images—and then added to each image in that data class. The perturbed/attacked images were then passed through the frozen pathology foundation model with its pre-trained linear classifier. Similar to the perturbation training method used in PSAP, the optimization of CSAP maximizes the cross-entropy between the model’s prediction and the one-hot ground-truth label over the class dataset (see the Methods). CSAP is highly effective within its desired scope, reducing the accuracy on its targeted class from 97.37% to 1.42% on training images and to 29.80% on unseen test images. Therefore, CSAP generalizes within its attacked data class and yet remains specialized, showing little effect on other tissue types/classes due to its design.

Compared to PSAP and CSAP, UTAP presents, by its design, the highest level of universality—a single, image-agnostic perturbation pattern applicable to almost all unseen samples. As summarized in Fig. 8, UTAP shows the strongest and most consistent generalization, yielding comparable degradation of classification accuracy on training (14.10%) and unseen test images (12.27%). Overall, as universality increases, overfitting diminishes, and the attack’s impact on novel images strengthens, making UTAP a practical and consequential threat.

### Universal and transferable adversarial attacks on pathology vision language models

To address whether the patch-level feature degradation induced by UTAP is sufficiently severe to undermine clinically relevant, slide-level workflows, we expanded our evaluations to encompass advanced pathology Vision-Language Models (VLMs) operating at both the patch and Whole Slide Image (WSI) levels. First, we evaluated the transferability of UTAP to a state-of-the-art patch-level pathology VLM (CONCH)^48^. As illustrated in Supplementary Fig. S8a, applying the fixed UTAP microscopic perturbation as an external attack successfully compromised the VLM’s frozen image encoder. Specifically, the adversarial patches were processed by the vision encoder and evaluated using a frozen, pre-trained linear classifier, resulting in a substantial reduction in linear classification accuracy from 95.6% to 78.9%. Furthermore, the attack fundamentally disrupted the multimodal representation space (Supplementary Fig. S8b). When the perturbed image features were correlated with text features extracted from class-specific text prompts (e.g., “An image of {class}”) via the frozen text encoder, the zero-shot image-text alignment accuracy significantly degraded from 79.1% to 69.8%, which further supports that the UTAP attack, injected only through in the visual domain, could damage the alignment between text and visual information, achieving success when attacking VLMs.

**Fig. 9:**
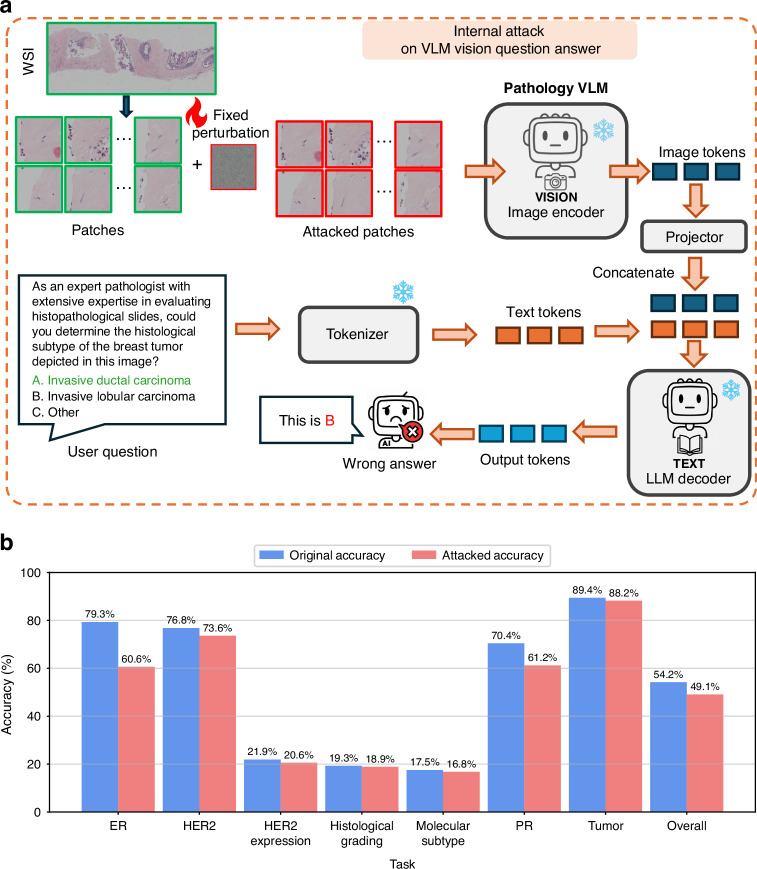
Universal adversarial attacks on pathology Vision-Language Models (VLMs). **a** Workflow of the internal attack on VLM visual question answering, illustrating the application of a fixed microscopic perturbation to WSI patches prior to feature extraction by the frozen image encoder and the LLM decoder. **b** The diagnostic accuracy across various clinical WSI-level tasks, with blue and red bars indicating the original and attacked accuracies, respectively

To further understand the universality and potential impact of UTAP on clinical workflows, we next investigate its effect on slide-level aggregation mechanisms for WSIs. For this, we evaluated the UTAP attack on SlideChat^49^, a large vision-language assistant designed for comprehensive WSI understanding. In this scenario (Fig. 9a), the fixed UTAP microscopic pattern was systematically added to the constituent image patches extracted from a WSI, prior to the foundation model-based feature extraction. Specifically, this perturbation was trained utilizing an identical methodology, directly targeting the CONCH model, which functions as the designated patch-level vision encoder for the VLM. These attacked patches were processed by the VLM’s frozen image encoder and a projection layer to generate image embedding tokens. These compromised visual tokens were subsequently concatenated with text tokens derived from a user diagnostic query (e.g., prompting the model to determine the histological subtype) and fed into a frozen Large Language Model (LLM) decoder. As detailed in our quantitative results (Fig. 9b), the corruption of these fundamental patch-level features was not smoothed out by the sequence aggregation; rather, it successfully propagated through the WSI-level architecture, undermining performance across a diverse set of clinical diagnostic tasks. Notably, the accuracy for Estrogen Receptor (ER) status prediction dropped from 79.3% to 60.6%, and Progesterone Receptor (PR) status declined from 70.4% to 61.2%. The overall diagnostic accuracy across all evaluated WSI tasks was systematically reduced from 54.2% to 49.1%. These findings provide compelling evidence that the microscopic adversarial disruptions introduced by UTAP are not mitigated by slide-level aggregation frameworks; instead, they accumulate to effectively compromise complex, WSI-level clinical predictions.

### Evaluation of spatial low-pass filtering defense and adaptive adversarial resilience

Given the profound vulnerability exposed by this highly generalizable threat posed by UTAP, it is imperative to explore potential countermeasures to secure pathology foundation models against such microscopic perturbations^50^. Because UTAP relies on a fixed, subtle pixel-level microscopic noise pattern to systematically corrupt the model’s feature space, an intuitive defensive strategy involves spatial filtering of the input images to neutralize the adversarial signals before they can be processed by the network. This motivated our investigation into standard spatial smoothing techniques.

**Fig. 10:**
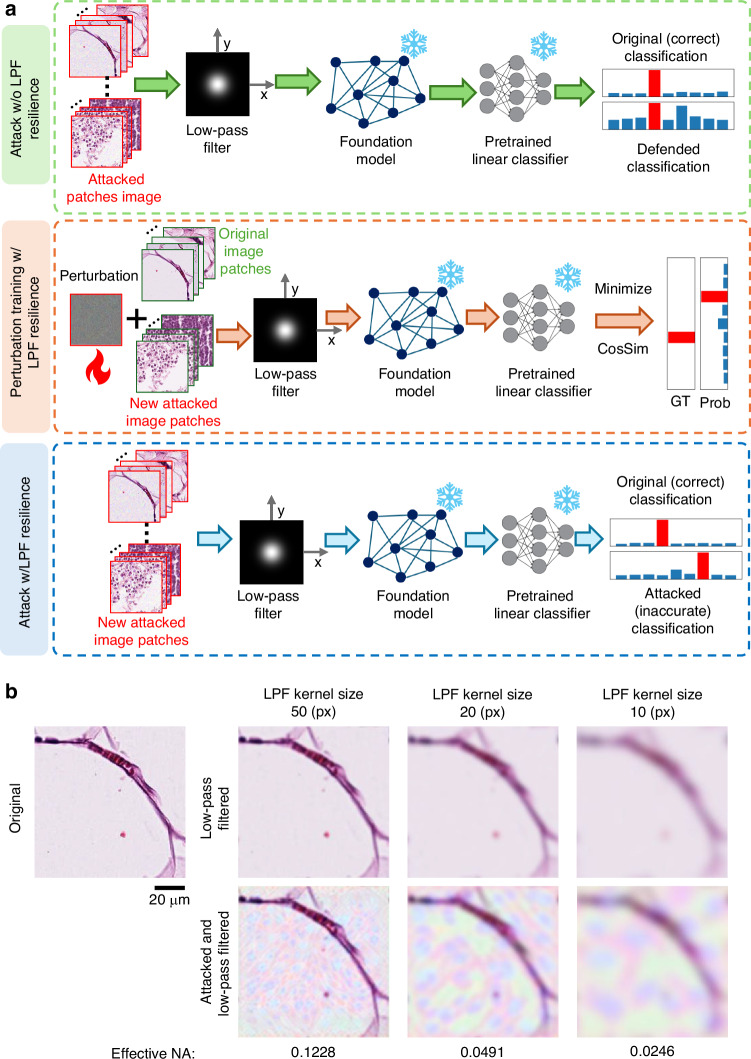
Spatial low-pass filter (LPF) defense and adaptive adversarial attacks with LPF resilience. **a** Workflows illustrating the LPF defense mechanism and the adaptive attack strategies with LPF resilience. The green dashed box shows the standard attack mitigated by the LPF; the orange dashed box details the perturbation training incorporated with LPF resilience, and the blue dashed box demonstrates the successful bypass of the defense by the adaptive attack. **b** Visual and physical impact of the LPF application on microscopic tissue images. The panels compare original, low-pass filtered, and attacked-and-filtered image patches across varying kernel sizes (50, 20, and 10 pixels), illustrating the corresponding degradation in morphological details and effective numerical aperture (NA)

To evaluate potential mitigation strategies against UTAP and assess the robustness of pathology foundation models, we investigated the application of a low-pass filter (LPF) as a defense mechanism prior to feature extraction. Because adversarial perturbations often manifest as high-frequency microscopic noise patterns, applying spatial smoothing could be a viable approach to neutralize such attacks. While applying an LPF to the attacked input image patches indeed mitigates the standard UTAP attack—effectively restoring the reduced downstream classification accuracy (see Fig. 10a, green dashed box)—implementing an LPF also introduces a severe and clinically unacceptable compromise in optical microscopic image quality. Physically, applying a low-pass filter to a microscopic image equates to a substantial reduction in the effective numerical aperture (NA) of the imaging system. As illustrated in Fig. 10b, decreasing the LPF kernel size to filter out adversarial noise progressively degrades the optical resolution, yielding effective NAs of ~0.123, ~0.0491, and ~0.0246 for kernel sizes of 50, 20, and 10 pixels, respectively. Because preserving high-fidelity morphological details is paramount for accurate histopathological diagnosis, artificially degrading the image resolution to defend against adversarial threats undermines the fundamental utility of the digitized histology slides.

**Fig. 11:**
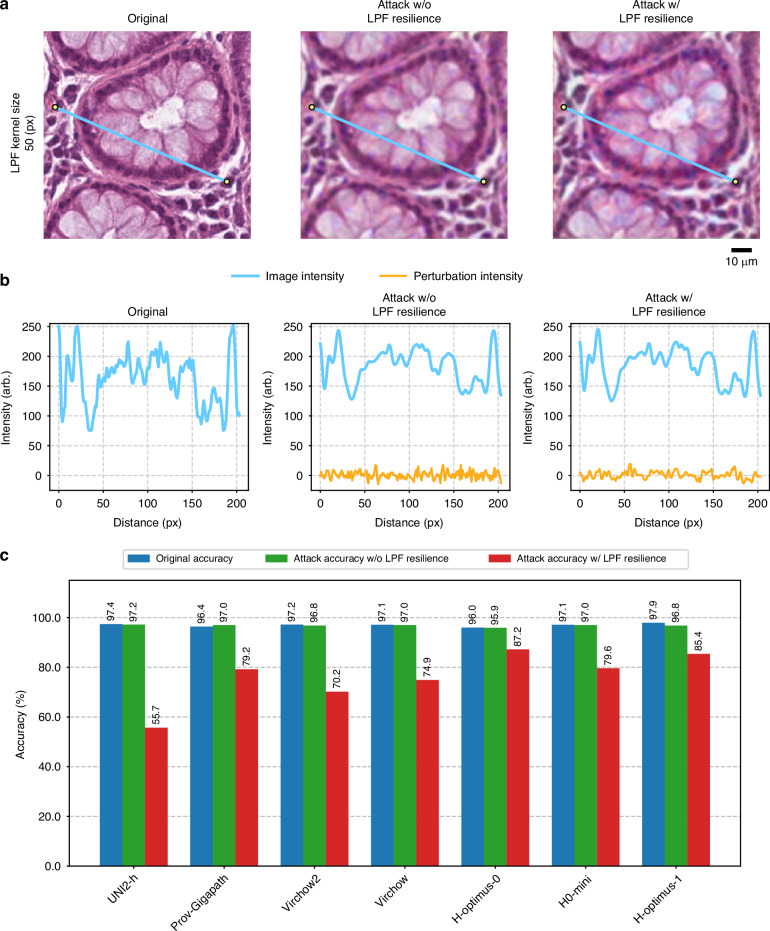
Bypassing low-pass filter defense using adaptive perturbations. **a** Microscopic image patches comparing the original tissue, the standard attack without LPF resilience, and the adaptive attack with LPF resilience. **b** Corresponding 1D spatial intensity profiles along the indicated cross-sections, demonstrating the low magnitude of the perturbation intensity (orange) relative to the underlying image intensity (blue). **c** Classification accuracy across seven foundation models evaluated under LPF defense. The blue bars indicate the original accuracy. The attacked classification accuracies *without* and *with* LPF resilience are shown with the green and red bars, respectively

Furthermore, we demonstrated that this LPF-based defense can be systematically bypassed by an adaptive adversary. To showcase this, we modified our UTAP optimization framework to incorporate the LPF into the forward pass during the perturbation training, thereby forcing the algorithm to minimize the feature-space cosine similarity under the assumption that an LPF-based defense is active (Fig. 10a, orange dashed box). As shown in the spatial intensity profiles where a 50-pixel LPF was applied (Fig. 11a,b), as well as the corresponding results for 20-pixel and 10-pixel kernel sizes (Supplementary Fig. S9), the LPF-resilient UTAP microscopic perturbations maintain a low magnitude, reserving the visual subtlety of the attack on the tissue structures. When the foundation models were subjected to this LPF-resilient attack (Fig. 10a, blue dashed box), the LPF defense was rendered highly ineffective. As shown in Fig. 11c, the classification accuracy dropped significantly across all evaluated foundation models despite the presence of the LPF-based defense. While the standard UTAP microscopic attack was largely mitigated by the LPF defense (green bars), the LPF-resilient UTAP attack (red bars) successfully bypassed this countermeasure, causing a severe drop in the classification accuracy of the internal model (UNI2-h). Furthermore, this degradation trend consistently transferred across all evaluated external black-box foundation models. Despite the active LPF defense, the LPF-resilient UTAP microscopic perturbations systematically corrupted the feature representations, leading to substantial accuracy reductions across the entire foundation model pool. These findings indicate that simple image-processing or spatial-filtering-based interventions are insufficient to secure computational pathology workflows, underscoring the necessity for deeper, architectural defense mechanisms, such as adversarial fine-tuning of foundation models or, eventually, human-in-the-loop quality assurance checks, which will be discussed next.

### Closed-loop methodology for the detection and analysis of adversarial attacks

**Fig. 12:**
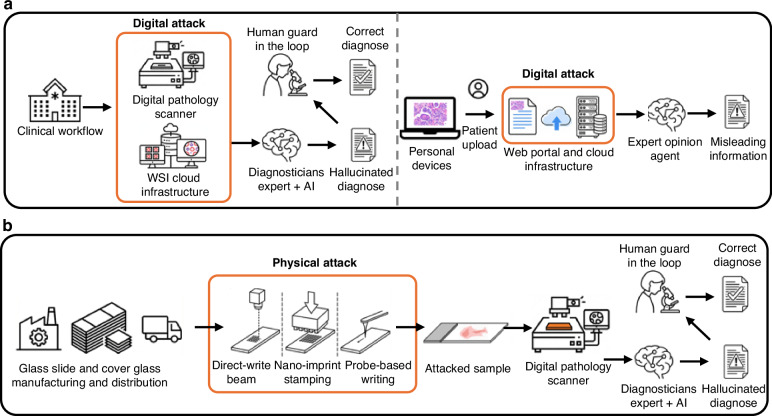
Realistic threat surfaces and injection points for adversarial attacks across the digital pathology pipeline. **a** Workflows illustrating digital-domain attacks occurring after optical image acquisition via software-centric compromises during whole-slide image transmission or storage in both standard clinical and remote settings. **b** Workflows illustrating physical domain attacks occurring prior to digital image acquisition via imperceptible nanoscale tampering with optical consumables (e.g., glass slides and cover slips) during manufacturing. In both attack domains, the inclusion of a ‘Human guard in the loop’ is highlighted as a critical strategy to reject hallucinated AI diagnoses and secure the correct clinical evaluation

To systematically defend against universal adversarial perturbations (UTAPs), it is essential to map their potential injection points across the optical-to-digital pathology pipeline. Vulnerabilities exist at virtually every stage, from the physical glass slide to the final classification result, encompassing optical image acquisition, data transmission to PACS (Picture Archiving and Communication System) and LIS (Laboratory Information System) archives, and downstream computational workflows—including preprocessing, feature embedding, and both patch- and slide-level classification tasks. As illustrated in Fig. 12, these potential threat surfaces can be categorized into two primary domains. First, digital-domain attacks occur after optical image acquisition via software-centric compromises that introduce fixed perturbations during WSI transmission or storage. In a standard clinical workflow, this attack surface includes digital slide scanners and associated WSI cloud infrastructure; in remote or second-opinion/tele-consultation scenarios, WSIs uploaded from personal devices can be intercepted or altered at the web portal level or during transmission, leading to misleading information. Second, physical-domain attacks can occur before digital image acquisition by imperceptibly tampering with optical consumables (e.g., tissue slide holders or coverslips). For example, during the manufacturing or distribution of glass slides and/or cover slips, adversaries can utilize nanoscale fabrication techniques to embed adversarial artifacts directly onto the physical substrate. Consequently, when the tissue is mounted, the scanner inherently digitizes the perturbation alongside the true biological morphology, which can contaminate the digitized images.

**Fig. 13:**
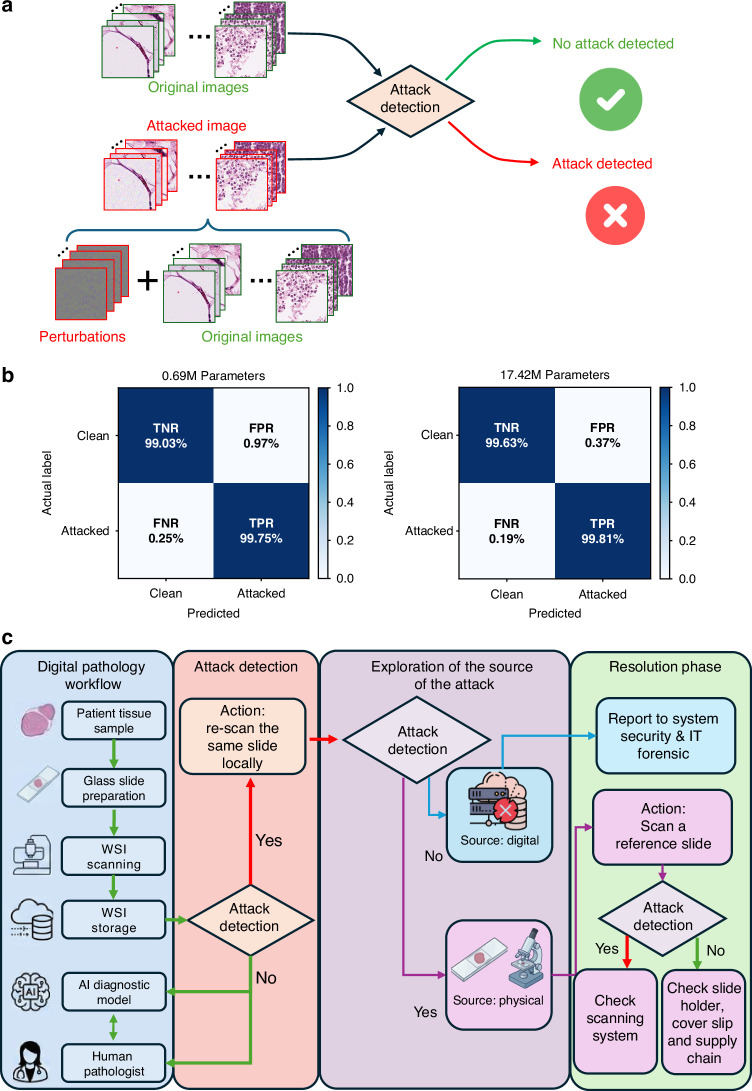
Closed-loop methodology for the detection, identification, and resolution of adversarial attacks. **a** Schematic of the attack detection process designed to distinguish between clean and perturbed microscopic images. **b** Confusion matrices demonstrating the high detection efficacy of both the light-weight (0.69 M parameters) and larger (17.42 M parameters) attack detection networks. **c** Flowchart detailing the systematic defense workflow deployed upon initial attack detection. The protocol triggers an exploration phase to localize the vulnerability source to either the digital infrastructure or the physical domain, guiding the subsequent systemic resolution and clinical reconfirmation actions

In both of these attack scenarios, the perturbation can successfully propagate to downstream foundation models, generating misleading information in fully automated systems or hallucinated diagnoses in collaborative AI workflows. To secure the clinical utility of pathology foundation models against these threats, a “*detection–identification–reconfirmation*” closed-loop methodology can be considered (see Fig. 13). Because UTAP-like attacks rely on fixed microscopic perturbations that induce consistent shifts in learned feature representations, initial detection can be achieved computationally. As a first line of defense, we developed a dedicated attack-detection network (reported in Fig. 13a) trained to identify the microscopic perturbations characteristic of such adversarial attacks. Evaluations of two network configurations demonstrated successful detection capabilities: a light-weight model (with 0.69 M parameters) achieved a True Negative Rate (TNR) of 98.69% and a True Positive Rate (TPR) of 99.66%, while a larger model (with 17.42 M parameters) achieved a TNR of 99.52% and a TPR of 99.78% as reported in Fig. 13b.

Upon detecting an attack during the standard digital workflow (e.g., at the WSI storage stage prior to clinical evaluation), the system can trigger an *exploration* phase to identify the source of the vulnerability (see Fig. 13c). The immediate reconfirmation action requires locally re-scanning the corresponding physical tissue slide. If the newly scanned WSI passes the detection check, the vulnerability can be localized to the digital infrastructure, prompting an immediate report to system security and IT forensics. Conversely, if the re-scanned local image continues to trigger attack detection, the source of the perturbation can be physical. Scanning a known *clean* reference tissue slide can be used to differentiate between a compromised microscopy scanning system (if the reference scan yields a positive attack detection) and a contaminated patient slide (if the reference scan is clear). Crucially, for definitive clinical reconfirmation and system rescue, this diagnostic workflow should integrate a “human guard in the loop.” While the visually subtle UTAP subverts the AI assistant, the attending diagnostician’s traditional morphological assessment remains unhindered. The resulting discordance between the compromised AI output and the human expert’s independent evaluation serves as the ultimate safeguard, potentially enabling the pathologist to flag the anomaly, reject the misleading information, and secure the correct diagnosis.

## Discussion

We presented UTAP, a single, universal perturbation that transfers across different pathology foundation models. In contrast to logit-level attacks that maximize cross-entropy to induce class flips along model-specific decision boundaries, UTAP intervenes earlier in the pipeline by directly disrupting the microscopic feature representations. Our training scheme is designed to disrupt a model’s core feature representations on classification/[CLS] tokens and also local patch features, yielding microscopic perturbations that are highly transferable and capable of compromising black-box foundation models on unseen images, *without* any model-specific or instance-specific post-training.

A central advantage of UTAP is its strong cross-model transferability demonstrated across various foundation models. UTAP targets the representation itself, corrupting a more fundamental and widely shared structure. During UTAP training, we optimize the microscopic perturbation using a cosine-similarity loss—rather than the cross-entropy objective used in PSAP and CSAP—to directly decorrelate the attacked feature vectors from the original representations. This objective drives the features to become maximally dissimilar, and compels the optimization to exploit the full spectrum of activations, including negative values, thereby reorienting the vectors in feature space. By manipulating the geometry of the representation manifold instead of merely disrupting logits across a decision boundary, UTAP induces a more fundamental corruption of representational power, which likely underpins its superior transferability across architectures and different black-box models. This effect is also evident in Fig. 2d–e: UMAP and PCA projections reveal a collapse of the feature manifold, preventing the formation of semantically meaningful clusters after the UTAP attacks. This strategy is particularly powerful against ViTs, which operate by dividing images into a grid of patches and learning local feature representations for each. By incorporating attention dropping and random masking regularization techniques during the training stage, the UTAP perturbation was forced to focus its optimization on disrupting these local, patch-level features rather than the more complex inter-patch relationships learned by the attention mechanism^37^, which resulted in a greater classification accuracy drop of external models as reported in Supplementary Fig. S3. The resulting grid-like pattern that is often visible in UTAP arises from its interaction with the ViT’s patch-embedding stage, where the perturbation aligns with position token boundaries and interacts with the model’s patch-based representation. To shed further light on this, we also present a high-contrast visualization of the microscopic perturbation in Supplementary Fig. S10, which clearly reveals these structural grid boundaries. The microscopic details of the UTAP perturbation often manifest as high-frequency, dark structures resembling scattered cell nuclei. The emergence of these nucleus-like features is a direct consequence of the optimization landscape in computational pathology. Because nuclear morphology and density are fundamental diagnostic entities in H&E-stained tissue, all state-of-the-art pathology foundation models universally learn to assign high attention weights to these structures during feature extraction. By iteratively minimizing the cosine similarity between the original and the attacked image embeddings, the UTAP optimization naturally converges to generate false, nucleus-like artifacts. These adversarial features act as universal distractors, effectively hijacking the self-attention mechanisms and corrupting the localized feature maps across different foundation models. Consequently, the high transferability of UTAP is driven by a dual vulnerability: the architectural patch-processing boundaries of the ViT backbone and the shared semantic reliance on nuclear morphology across all pathology foundation models.

To investigate the influence of the training set size on the attack’s efficacy, we conducted an ablation study by varying the number of images ($N$) used to optimize UTAP. As shown in Supplementary Fig. S11, increasing the number of training images from $N=900,\,1800,\,2700$ to $N=100k$ (full training dataset) yielded no significant improvement in the attack’s performance across both internal and external foundation models. Post-attack classification accuracies were largely consistent across settings with small variations. This suggests that a modest, diverse image dataset suffices to capture generalizable vulnerabilities of pathology foundation models in representation space. To conserve computation, we therefore used $N=900$ in our results unless otherwise noted; the training time for a single UTAP pattern on 900 training samples took ~13 min.

To further confirm that the efficacy of our attacks stems from UTAP’s optimized delicate structure rather than the mere addition of noise, we conducted an ablation study using a randomly generated noise perturbation of the same intensity level. For this experiment, we generated a random noise pattern with pixel values uniformly sampled within the same magnitude constraint of $\epsilon =20$ used for UTAP. As illustrated in Supplementary Fig. S12, adding this unstructured random noise to the input images had a negligible effect on the performance of all the tested foundation models. The classification accuracy remained high across the board, with only a minimal drop observed. This result demonstrates that the foundation models are inherently resilient to unstructured, random perturbations of this magnitude. It further underscores that the potency of UTAP is not simply a consequence of its energy or the magnitude of the microscopic noise added but is derived from its carefully crafted, adversarial structure, which is specifically optimized to disrupt the fundamental feature representations across pathology foundation models.

While this work provides a comprehensive analysis of universal and transferable attacks, our investigation centered on the vulnerabilities of ViT-based foundation models, which are currently the dominant design choice in almost all visual foundation models. As detailed in the Results section, in order to validate the broader applicability and cross-architecture transferability of the UTAP framework, we also extended our evaluation to established CNN-based pathology models, including KimiaNet^45^ and CSCO^46^. By applying the single, fixed UTAP microscopic perturbation—originally optimized on a ViT backbone—directly to images processed by these CNN models, we observed significant performance degradation across a diverse array of datasets (see Supplementary Fig. S6). These results demonstrate that the fundamental feature representation collapse induced by UTAP is not strictly bound to the spatial tokenization mechanism unique to ViTs. Instead, the perturbation acts as a highly transferable, universal feature distractor that can compromise the global feature aggregation of different neural network architectures. Extending this framework to evaluate hybrid models remains a compelling direction for future work.

Our evaluations in this work were primarily focused on the downstream task of tissue classification on the image patch-level. Our approach can be further extended to other pathology tasks, such as semantic segmentation^29,51^ and cell counting^31^, to understand how these universal perturbations impact models’ ability to perform fine-grained spatial analysis and quantification. Broadening the investigation across these dimensions will be essential for developing a truly holistic understanding of AI safety in digital pathology and optical microscopy. Furthermore, it is crucial to investigate the impact of adversarial microscopic perturbations on WSI-level tasks. Many slide-level diagnostic models operate with weakly-supervised conditioning and aggregate features from numerous patches in different ways. Most opt to use only feature vectors of their [CLS]/classification tokens through multi-instance learning^52^, cascaded transformers^29,31^ or other architectures like LongNet^53^. As demonstrated by our expanded evaluations on advanced pathology VLMs, the patch-level feature degradation induced by UTAP is severe; the attack fundamentally disrupts the multimodal representation space, degrading zero-shot image-text alignment without requiring architecture-specific adaptation. Crucially, when applied to large vision-language assistants designed for WSI understanding, the corruption of fundamental patch-level features is not smoothed out by sequence aggregation. Instead, the adversarial disruptions successfully propagate through the WSI-level architecture, accumulating to effectively compromise clinical diagnostic predictions. These findings confirm that, because the proposed attack successfully distorts fundamental patch-level representations, the performance of any multi-scale or WSI-level model that depends on them will inevitably degrade.

The rapid creation of a universal and transferable perturbation pattern, in less than 15 min of training time, carries significant implications for the clinical deployment and safety of AI in digital pathology and optical microscopy. The demonstrated success of a transferable, black-box attack indicates that even proprietary—and potentially FDA-regulated—diagnostic systems can be compromised without access to the model, posing a direct risk to patient safety. This vulnerability highlights the need for mandatory adversarial-robustness evaluations and watchdog development efforts prior to deployment, extending beyond standard accuracy metrics to ensure resilience against sophisticated threats. Crucially, this work provides both a rigorous benchmark and a practical pathway to defense. UTAP serves not only as a stress test for foundation model robustness but also as a training signal for hardening models in practice. By incorporating UTAP-generated perturbations into adversarial training^54,55^, developers can effectively “vaccinate” models against a broad class of stealthy, transferable attacks. This strategy can potentially strengthen intrinsic safety, support regulatory confidence, and advance the reliable clinical adoption of AI in digital pathology and microscopy.

To further contextualize the threat posed by the UTAP, we also investigated the efficacy of advanced, feature-level defense mechanisms, specifically the integration of robustness tokens^56^. For this analysis, robustness tokens were trained separately and independently for each foundation model to ensure a tailored defensive baseline. Our evaluations revealed that while this mechanism successfully preserves model performance on clean, unperturbed data—yielding negligible discrepancies such as a minor shift from 97.4% accuracy to 97.3% for the UNI2-h model and 96.4% to 97.2% for the Prov-Gigapath model—it offers significantly limited protection against UTAP. On the source model (UNI2-h), where the adversarial perturbation was originally optimized, the defense provided only marginal recovery, with the classification accuracy slightly improving from 12.3% under the undefended attack to 14.4% with the defense active. More critically, when evaluating transferability to external models, the implementation of robustness tokens paradoxically accelerated the adversarial degradation in certain architectures. For instance, the attack accuracy of the Virchow model dropped further, from 24.5% to 19.9%, when the defense was applied, which defeats the purpose of the defense. This exacerbation was most pronounced in the Prov-Gigapath model: while the standard UTAP reduced its accuracy to 48.7%, the activation of its independently trained robustness tokens exposed a profound security gap, causing a catastrophic collapse that plummeted the accuracy to a mere 9.96%, defeating the purpose of this defense mechanism once again. These findings suggest that incorporating supplementary defensive parameters may inadvertently introduce new architectural vulnerabilities, exacerbating the feature space collapse when the model is confronted with highly optimized, representation-level perturbations.

## Materials and methods

### Adversarial attack dataset and pathology foundation models

The primary dataset used in this study is the NCT-CRC-HE-100K (short as CRC-100K)^35^, a collection of 100,000 non-overlapping image patches from H&E stained histological images of human colorectal cancer and normal tissue. All images are 224 × 224 pixels at a resolution of 0.5 µm per pixel with a 20x objective and were color-normalized using the Macenko’s^57^ method. The dataset comprises nine manually annotated tissue classes: Adipose (ADI), background (BACK), debris (DEB), lymphocytes (LYM), mucus (MUC), smooth muscle (MUS), normal colon mucosa (NORM), cancer-associated stroma (STR), and colorectal adenocarcinoma epithelium (TUM). These images were manually extracted from 86 H&E-stained formalin-fixed paraffin-embedded (FFPE) whole slides from the NCT Biobank and the UMM pathology archive, which included CRC primary tumors, liver metastases, and normal tissue regions from gastrectomy specimens to increase variability. For validation, an independent test set, CRC-VAL-HE-7K^36^, was used, containing 7180 image patches from 50 patients with colorectal adenocarcinoma who were not part of the NCT-CRC-HE-100K (training) cohort. In the further evaluation of UTAP’s universality (shown in Supplementary Fig. S4) on external datasets, we used the TCGA Uniform Tumor dataset^40^, comprising 1,608,060 image patches extracted from H&E-stained histological samples across 32 solid tumor types. To establish a simplified testbed, we restricted the label space to a subset of six representative classes—Adrenocortical, Bladder, Cervical, Esophageal, Kidney, and Mesothelioma. Patches from the TCGA Uniform Tumor dataset, originally sized 256 × 256 pixels, were center-cropped to 224 × 224 to match the input dimensions of the pre-optimized UTAP.

We primarily used a foundation model pool containing the following seven pathology foundation models directly accessed from HuggingFace: [*MahmoodLab/UNI2-h, prov-gigapath/prov-gigapath, paige-ai/Virchow2, paige-ai/Virchow, bioptimus/H-optimus-0, bioptimus/H0-mini, bioptimus/H-optimus-1*]^29–35^. While these models share the foundational ViT backbone to achieve state-of-the-art performance across clinical downstream tasks, they have diverse structural hyperparameters and are individually trained on different large-scale datasets. All the models have the vision transformer architecture as backbone, achieving state-of-the-art performances across multiple clinical downstream tasks in histopathology. A typical ViT forward pass partitions an image into non-overlapping patches, flattens each patch, and projects it with a trainable linear layer to obtain patch embeddings. A learned classification token ([CLS])^50^ is first prepended to the sequence, and the learned positional embeddings are later added. The sequence then passes through a stack of Transformer encoder blocks, each composed of multi-head self-attention (via query, key, and value projections) and a multiple-layer perceptron (MLP), with LayerNorm and residual connections^58^. While our experiments aim at attacking foundation models using ViT architectures with a 14-pixel patch size as the default design choice, the approach is broadly applicable to ViTs with different patch encoding schemes, including patch sizes of 16 or 28 pixels.

### Adversarial Microscopic Perturbation Training and Evaluation

In UTAP training, we implemented an adaptive projected gradient descent algorithm to optimize the perturbation by traversing through the training dataset $L$ times/epochs. The trainable perturbation ($p$) was first initiated as all zero values. At each optimization step, a new patch-level binary mask $M\in {\left\{\mathrm{0,1}\right\}}^{224\times 224}$ with the same spatial size as the input image was generated (denoted as RandomMasking()). To construct *M*, the 224 × 224 mask is partitioned into a 16 × 16 grid of non-overlapping 14 × 14 tiles (256 tiles total, same as the ViT patch embedding dimension). We then randomly select, uniformly and without replacement, a set of ${n}_{M}$ tile indices and set the pixels inside those tiles to 1 (all other pixels are 0). Unless noted otherwise, we use ${n}_{M}=130$ ( ≈ 51% of tiles). The binary mask was pixel-wise multiplied (denoted as $\odot$) to the trainable perturbation. This masked perturbation ($p\odot M$) was added to a batch ($B$) of original/clean training images (${I}_{{ori}}$) sampled from the training dataset (denoted as ${\mathscr{D}}$), *B* out of *N* images, to create the attacked image patches ${I}_{{atk}}={I}_{{ori}}+{p}\odot M$; see *Algorithm 1*. The original and attacked image patches were both normalized by the preprocess functions (denoted as ${\mathscr{T}}$) provided by each foundation model and then passed through the pathology foundation model backbone (denoted as ${\mathscr{M}}$) to extract the original (${f}_{{ori}}$) and attacked (${f}_{{atk}}$) embedding features (CLS tokens). The cosine similarity ($\mathrm{CosSim}(\cdot )$) between ${f}_{{ori}}$ and ${f}_{{atk}}$ was then calculated and minimized using the adaptive PGD method with perturbation bound $\epsilon$ and step size $\alpha =\frac{\epsilon \theta B}{255\times {LN}}$ where $\epsilon$ is the maximum L-infinity norm of the perturbation and $\theta$ is a hyperparameter that controls the scaling factor of each PGD step size. During the adaptive PGD backpropagation steps, the gradient of the self-attention operations of the transformer blocks was set to zero to force the UTAP to better focus on the local feature representation capability of the foundation models rather than cross-token correlation to enhance the transferability of the adversarial attack, same as Pay No Attention (denoted as $\mathrm{PNA}(\cdot )$)^37^. The $\mathrm{Clamp}(\cdot )$ function limits the input value to a range between a minimum and a maximum value by setting out-of-bound values to the nearest bound. We formalize the UTAP training algorithm in Table 1, reported below.

**Table 1:**
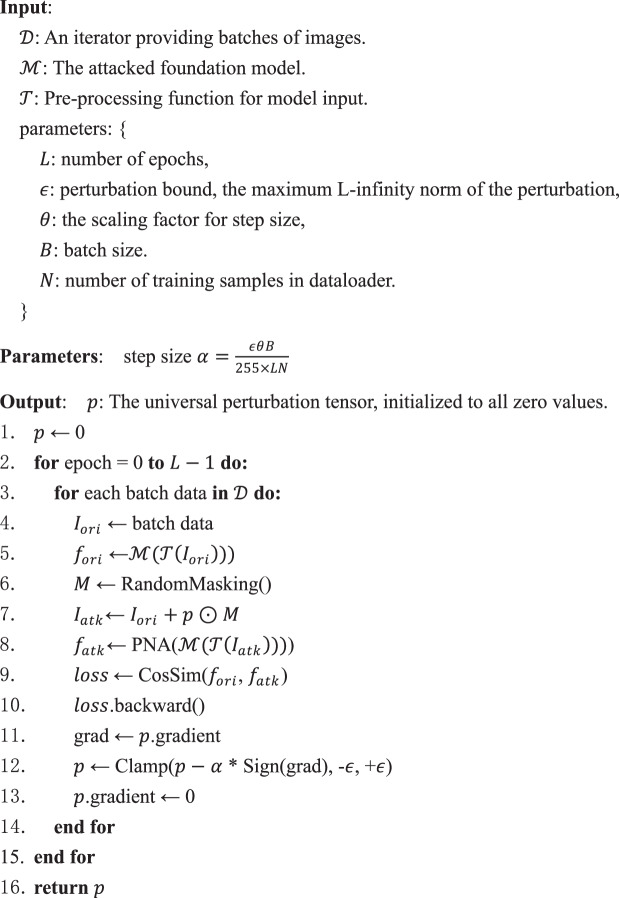
Training Algorithm of UTAP

The UTAP pattern, added to new image patches (clipped to [0, 255] to avoid overflow), was evaluated both internally (same foundation model as used in training) and externally (on unseen black-box foundation models). The attacked classification accuracy was evaluated by calculating the match between the ground truth label and the final prediction from the attacked images. The dropped accuracy was calculated by the difference between the classification accuracy of the original images and the attacked images.

In the PSAP training, patch-specific perturbations were added to the original images to create the attacked images, which passed through the frozen foundation model and the frozen pretrained linear classifier to get the classification probabilities. The cross entropy between the classification probabilities and the label ground truth was maximized to optimize the patch-specific perturbation with a total number of 100 steps and a step size of 0.01 for each perturbation. Although each perturbation was trained on a single image, we evaluated the PSAP attack on both the trained images and new/testing image patches. The attacked classification accuracy and accuracy drop were calculated the same way as in the evaluation of UTAP. In the CSAP training, we develop one perturbation for each target data class, i.e., all images in the same class share the same attack pattern. Similar to PSAP, we aimed to maximize the cross entropy in the optimization process. The CSAP attack was also evaluated by adding the class specific perturbation to the corresponding trained and new image patches, which included images both inside and outside the target class. The attacked classification accuracy and the accuracy drop were calculated in the same way as in the evaluation of UTAP.

To establish a baseline comparison, we also implemented the Fast Gradient Sign Method (FGSM)^39^. In the FGSM attack, original images were passed through the frozen foundation model and the pre-trained linear classifier to compute cross-entropy loss against the ground-truth label. Image-specific perturbations were then generated in a single step by extracting the sign of the loss gradient with respect to the input pixels and scaling it by a predetermined magnitude. These perturbed images were evaluated, and the attacked classification accuracy and accuracy drop were calculated in the same manner as in the evaluation of UTAP.

### Visualization of adversarial perturbations

To effectively visualize the structure of the adversarial perturbation, we superimposed the perturbation onto a uniform gray background (R, G, B values: [128, 128, 128]). This method makes subtle, high-frequency patterns of perturbation visible against a neutral gray image. For histogram analysis, the perturbation’s RGB channels were averaged to create a single-channel intensity map. The histogram was then computed from the pixel intensity distribution of this grayscale representation.

### Training loss functions and backend classifier training

The UTAP patterns were optimized using adaptive projected gradient descent-based supervised learning methods, which minimized the cosine similarity of the original and attacked features extracted by the frozen pathology foundation models as detailed in *Algorithm 1*. The cosine similarity was defined as:

1 $$\mathrm{CosSim}\left({f}_{{ori}},{f}_{{atk}}\right)=\frac{{f}_{{ori}}\cdot {f}_{{atk}}}{{||}{f}_{{ori}}{||}\cdot {||}{f}_{{atk}}{||}}$$

where ${||}\cdot {||}$ calculates the second-order norm of a vector, ${f}_{{ori}}$ and ${f}_{{atk}}$ denote the original and attacked features vectors which correspond to the classification**/**[CLS] token—a global feature aggregator introduced in the Transformer^59^ architecture—extracted from the frozen pathology foundation model. The dimensionality of these feature vectors is dependent on the specific foundation model employed. The PSAP and CSAP were optimized using the cross entropy of ${\rm{Softmax}}$-activated classification logits calculated by the pretrained linear classifier and the ground truth label, defined as:

2 $$\mathrm{CE}\left({gt},{logits}\right)=-{\sum }_{i}^{C}g{t}_{i}\cdot \log (\mathrm{Softmax}{\left({logits}\right)}_{i})$$

where ${gt}$ is the one-hot encoded ground truth label, ${logits}$ is the output of the pretrained linear classifier, $C$ is the total number of classes, and $\mathrm{Softmax}(\cdot )$ was calculated by:

3 $${\rm{Softmax}}{\left(x\right)}_{i}=\frac{{e}^{{x}_{i}}}{{\sum }_{j}^{C}{e}^{{x}_{j}}}$$

where ${x}_{i}$ is the i-th logit from the linear classifier’s prediction. Note that for PSAP and CSAP training, we maximized cross entropy.

For each foundation model, a logistic regression model serves as the backend linear classifier^60^. These classifiers were trained on precomputed features from the original training image patches and evaluated on features from a separate test dataset, with the foundation model backbone frozen. The logistic regression classifiers’ weights were optimized using the L-BFGS^61^ algorithm to minimize a composite objective function comprising the cross-entropy loss and an L2 regularization term. The optimization process utilized a strong Wolfe line search^62^ and was run for a maximum of 100 iterations. A typical training time for a linear classifier is less than 2 s.

### Parameters of digital implementation and training scheme

For UTAP training, $N=900$ image patches of $224\times 224$ pixels were sampled from the training dataset with 100 images per class. The original and attacked images were normalized using the same methods as the corresponding foundation model was trained with. UTAP was optimized for $L=10$ epochs, iterating by $B=5$ images per batch. The perturbation was updated using adaptive PGD methods with $\theta =10$, step size $\alpha =\frac{\epsilon \theta B}{255\times {LN}}=4.35\times {10}^{-4}$ and clamped to the range between $[-\epsilon ,\epsilon ]$ where $\epsilon =20$ after each iteration.

The numerical simulations and the training process for UTAP were carried out using Python (version 3.11.15) and PyTorch (version 2.1.2, Meta Platform Inc.). The UTAP optimization underwent a 10-epoch training on a workstation equipped with an Nvidia GeForce RTX 4090 GPU, an Intel Core i9-13900KF CPU, and 32 GB RAM. The training time for a single UTAP ($224\times 224\times 3$ pixels) on 900 samples was ~13 min.

## Supplementary information

Supplementary Information

## Data Availability

All the data and methods needed to evaluate the conclusions of this work are presented in the main text and Supporting Information. The codes used in this work use standard libraries and scripts that are publicly available in PyTorch, and they can be accessed through: https://drive.google.com/drive/folders/1_-WjU-Rw6pmOG3wtNQtn2y9OfeeacPu4?usp=drive_link
