# Supplementary material for: Universal and transferable attacks on pathology foundation models using microscopic perturbations
Source: Light Sci Appl. 2026 Jun 1;15:258. doi: 10.1038/s41377-026-02347-w (PMC13226705; doi:10.1038/s41377-026-02347-w)
Supplement: Supplementary file 1 — Supplementary Information [file 41377_2026_2347_MOESM1_ESM.pdf]

Supplementary Information for

Universal and Transferable Attacks on Pathology Foundation Models Using Microscopic Perturbations

Yuntian Wang[1,2,3†], Xilin Yang[1,2,3†], Che-Yung Shen[1,2,3], Shuhang Dong[4], Nir Pillar[5] and Aydogan Ozcan[1,2,3,6*]

[1]Electrical and Computer Engineering Department, University of California, Los Angeles, CA, 90095, USA

[2]Bioengineering Department, University of California, Los Angeles, CA, 90095, USA

[3]California NanoSystems Institute (CNSI), University of California, Los Angeles, CA, 90095, USA

[4]Department of Mathematics, University of California, Los Angeles, CA, 90095, USA

[5]Department of Pathology, Hadassah Hebrew University Medical Center, Jerusalem, 91120, Israel

[6]Department of Surgery, University of California, Los Angeles, CA, 90095, USA

[†]These authors contributed equally to the work

[*]Correspondence to: ozcan@ucla.edu

This PDF file includes:

Supplementary Figures S1-S12

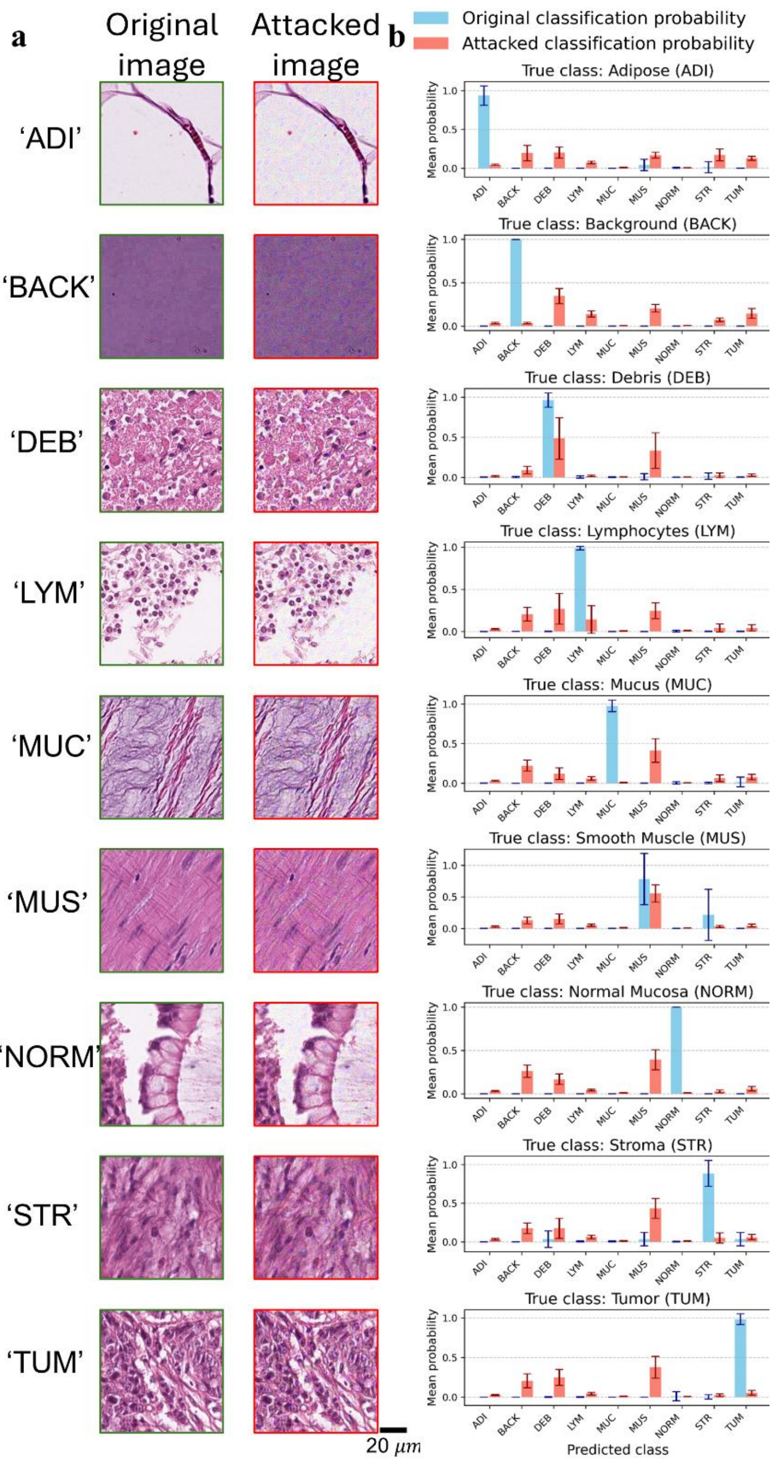

Supplementary Fig. S1. Classification probabilities of the original and the attacked microscopic images of different classes. (a) The original and the attacked images of the 9 different classes from the CRC-100K dataset[1]. (b) The classification probabilities of the original (orange) and the attacked images

(blue) predicted by a linear classifier trained by the [CLS] token extracted from the internal model (UNI2-h[2]).

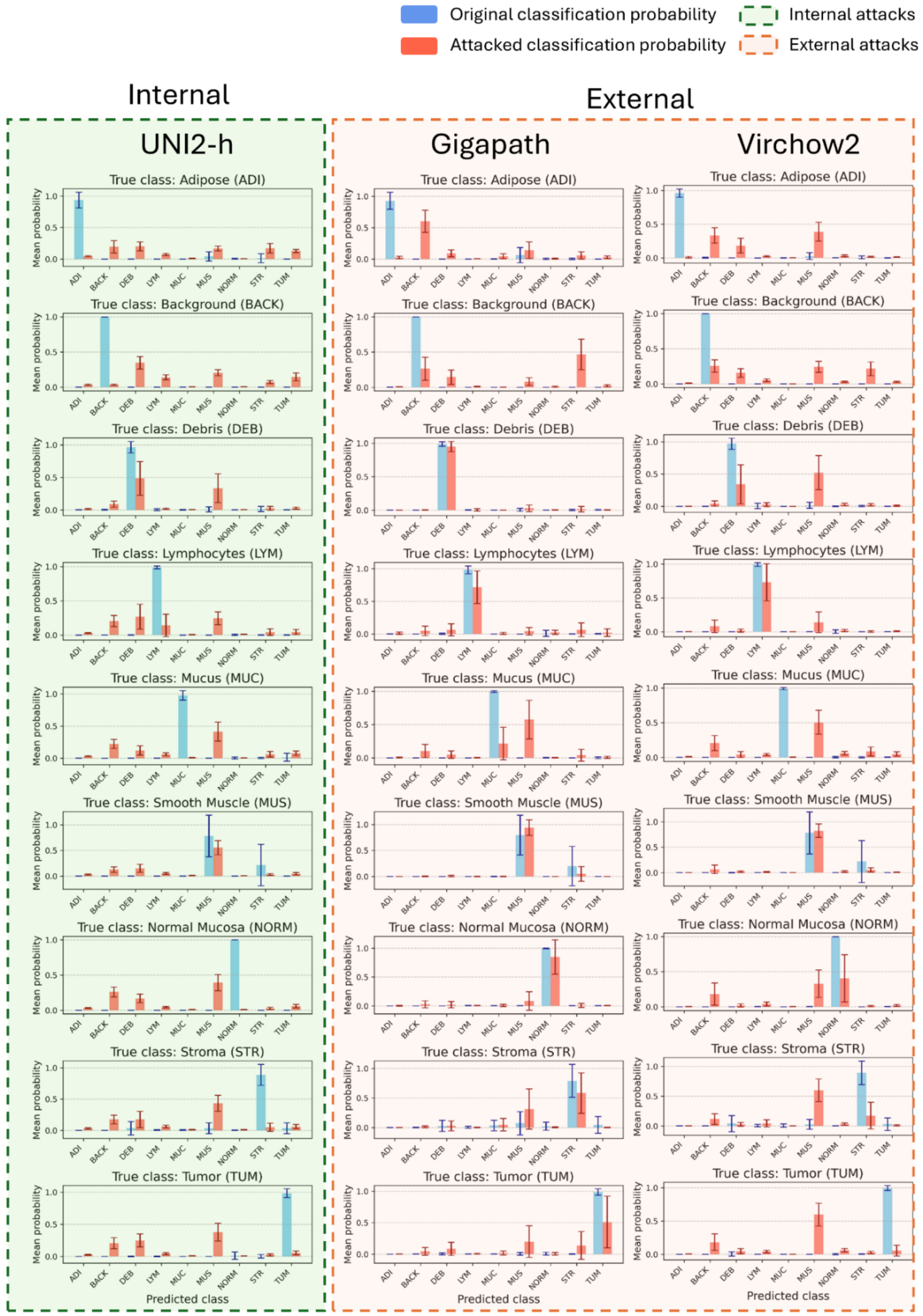

Supplementary Fig. S2. Classification probability comparison of the internal (UNI2-h) and external foundation models (Gigapath[3] and Virchow2[4]). The classification probabilities of the original (orange bars) and the attacked images (blue bars) are predicted by a linear classifier trained by the [CLS] token

extracted from the corresponding model. The evaluation of internal and external models is shown in the dashed green and orange boxes, respectively.

a

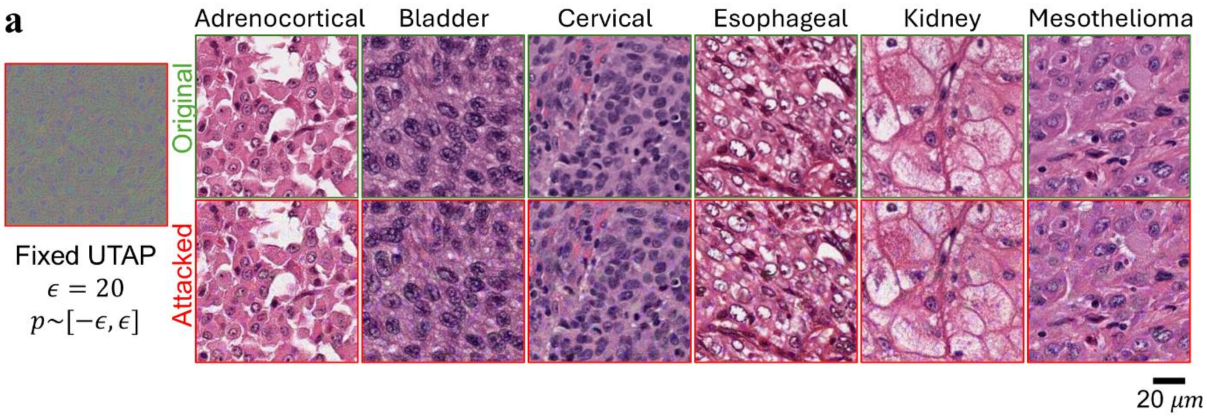

b

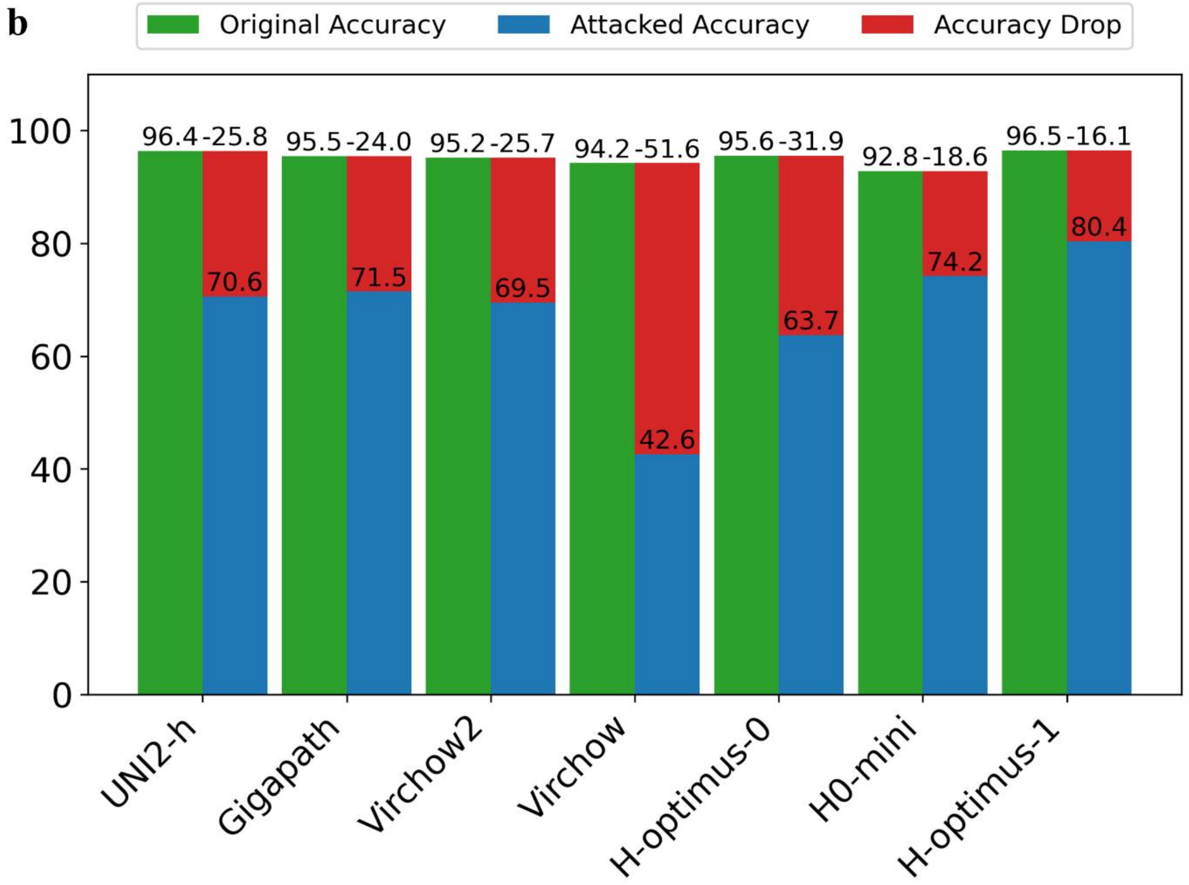

Supplementary Fig. S3. UTAP attack results on the external TCGA Uniform Tumor dataset[5]. (a) Visualization of the optimized UTAP with the original and the attacked images of six classes sampled from the reduced TCGA Uniform Tumor dataset. (b) The original (green), attacked (blue) and dropped (red) classification accuracy values on seven different pathology foundation models.

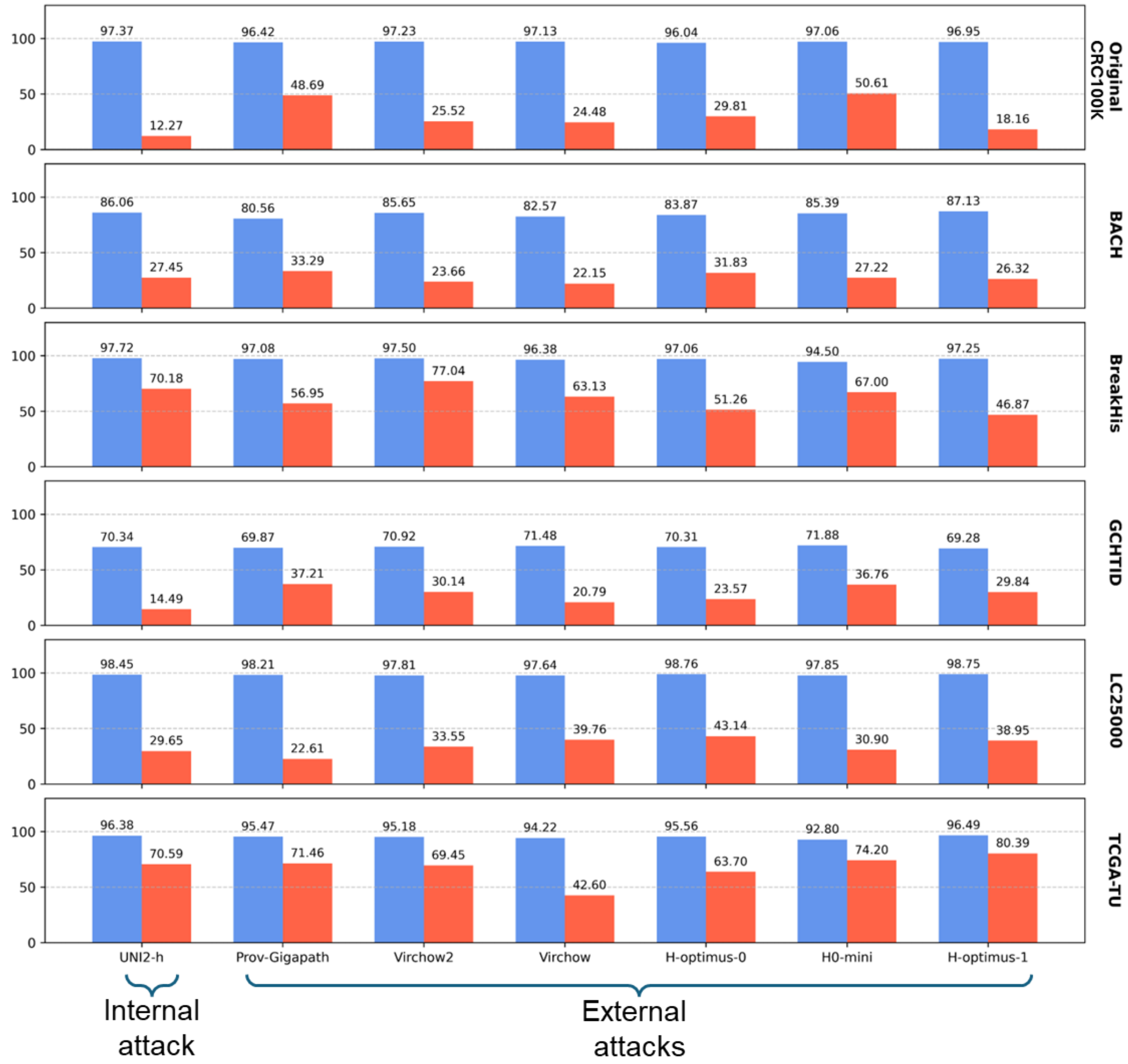

Supplementary Fig. S4. Generalization of UTAP across out-of-distribution datasets. Classification accuracies of seven pathology foundation models evaluated on the original test distribution (CRC100K) and five unseen external datasets (BACH[6], BreakHis[7], GCHTID[8], LC25000[9], and TCGA-TU[5]). Blue and orange bars indicate the original and attacked classification accuracies, respectively.

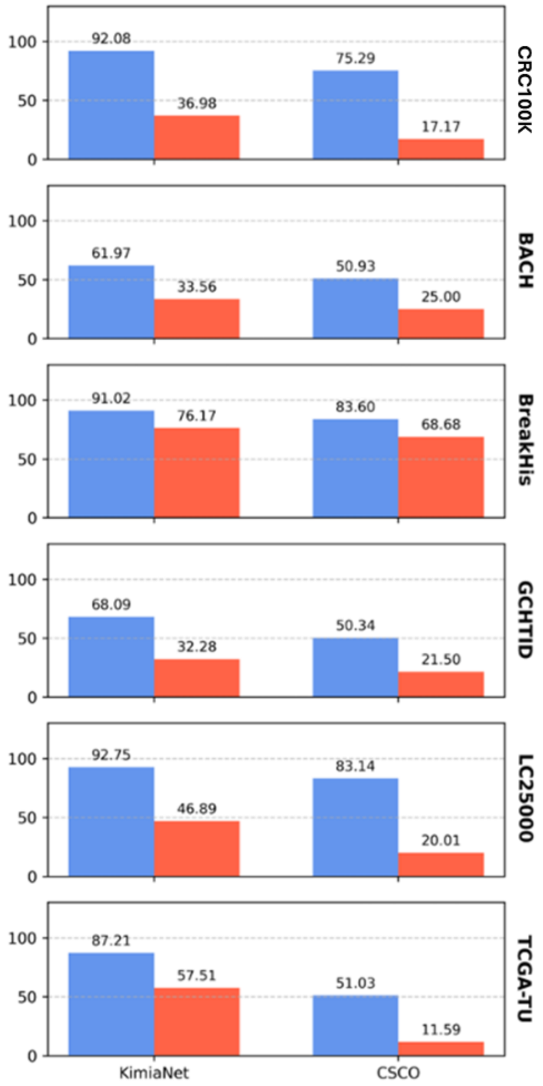

Supplementary Fig. S5. Generalization of UTAP across out-of-distribution datasets for unseen CNN-based models. Classification accuracies of two CNN-based foundation models (never seen during training) were evaluated on the original test distribution (CRC100K) and five unseen external datasets (BACH[6], BreakHis[7], GCHTID[8], LC25000[9], and TCGA-TU[5]). Blue and orange bars indicate the original and attacked classification accuracies, respectively.

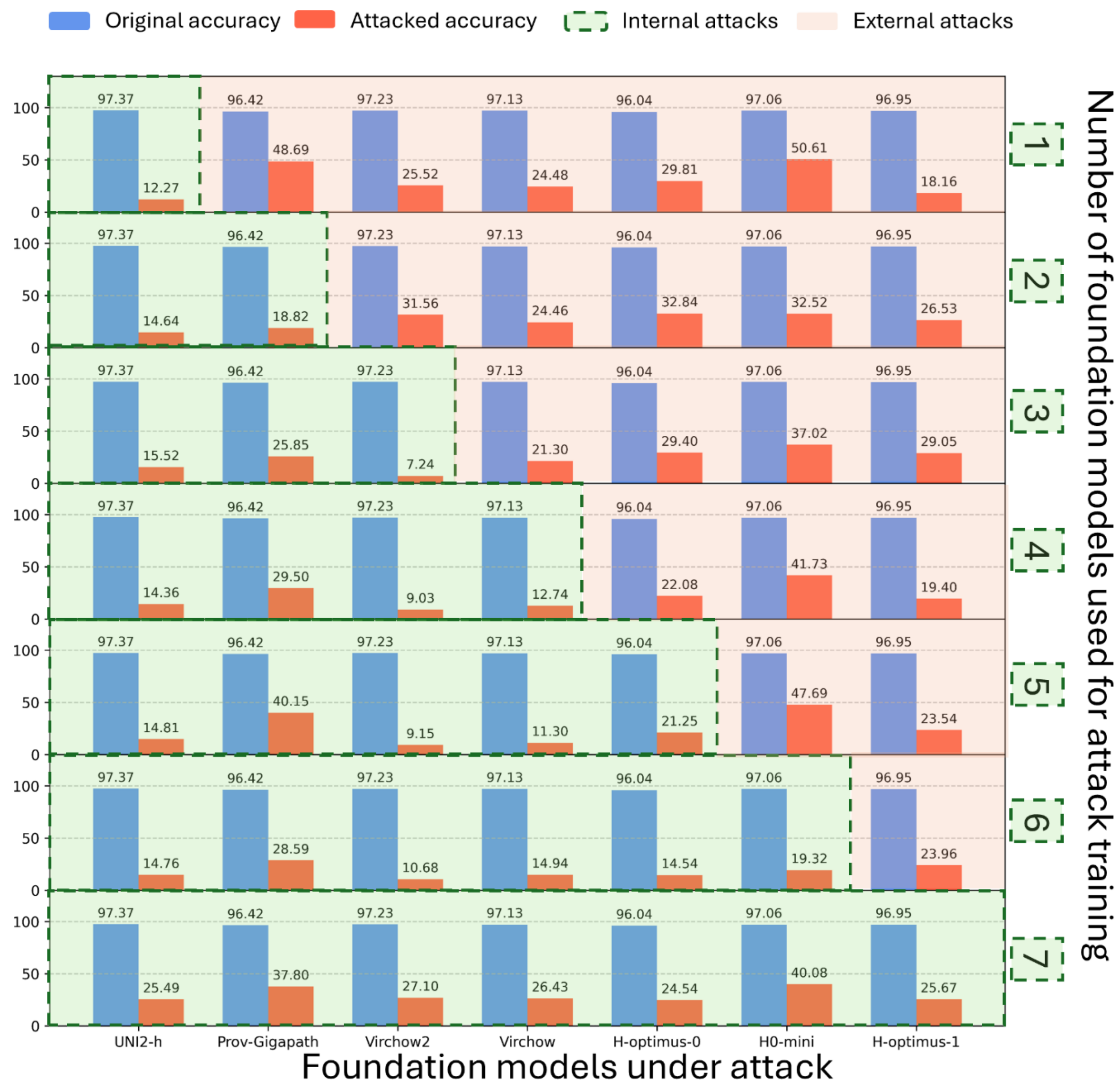

Supplementary Fig. S6. Cross-model transferability of UTAP trained by different numbers of foundation models. The matrix displays the attack performance of UTAP perturbations trained on different numbers of foundation models and evaluated against all the foundation models, including the internal (green dashed boxes) and external (orange boxes) models.

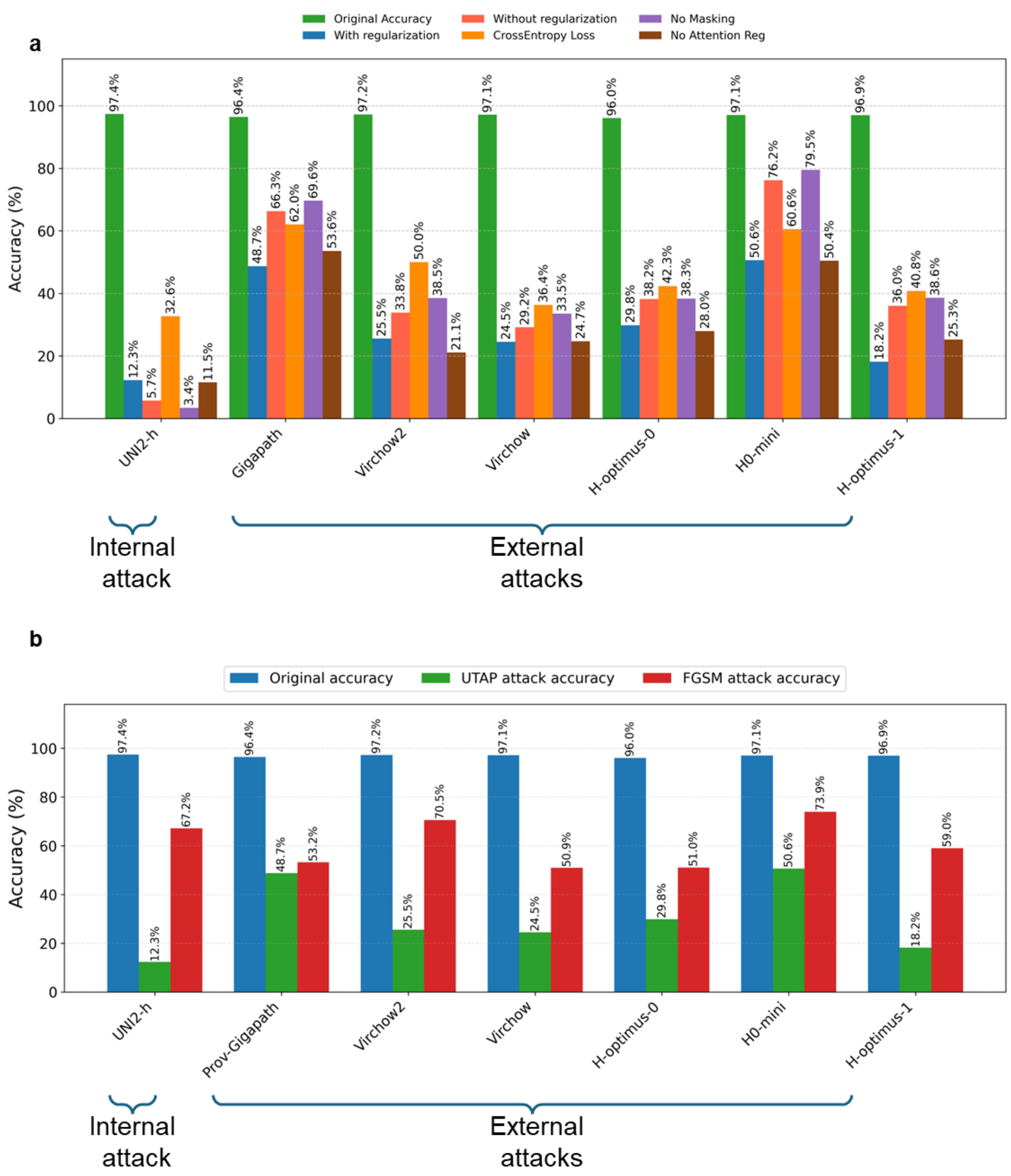

Supplementary Fig. S7. Ablation studies and baseline comparison of the UTAP framework. (a) Classification accuracies of seven foundation models, evaluating the impact of different optimization objectives (e.g., cross-entropy loss) and regularization components (e.g., attention dropping and random masking) on the efficacy of internal and external attacks. (b) Performance comparison between the UTAP

method and the Fast Gradient Sign Method (FGSM), illustrating the superior degradation capability and transferability of UTAP across all the evaluated models.

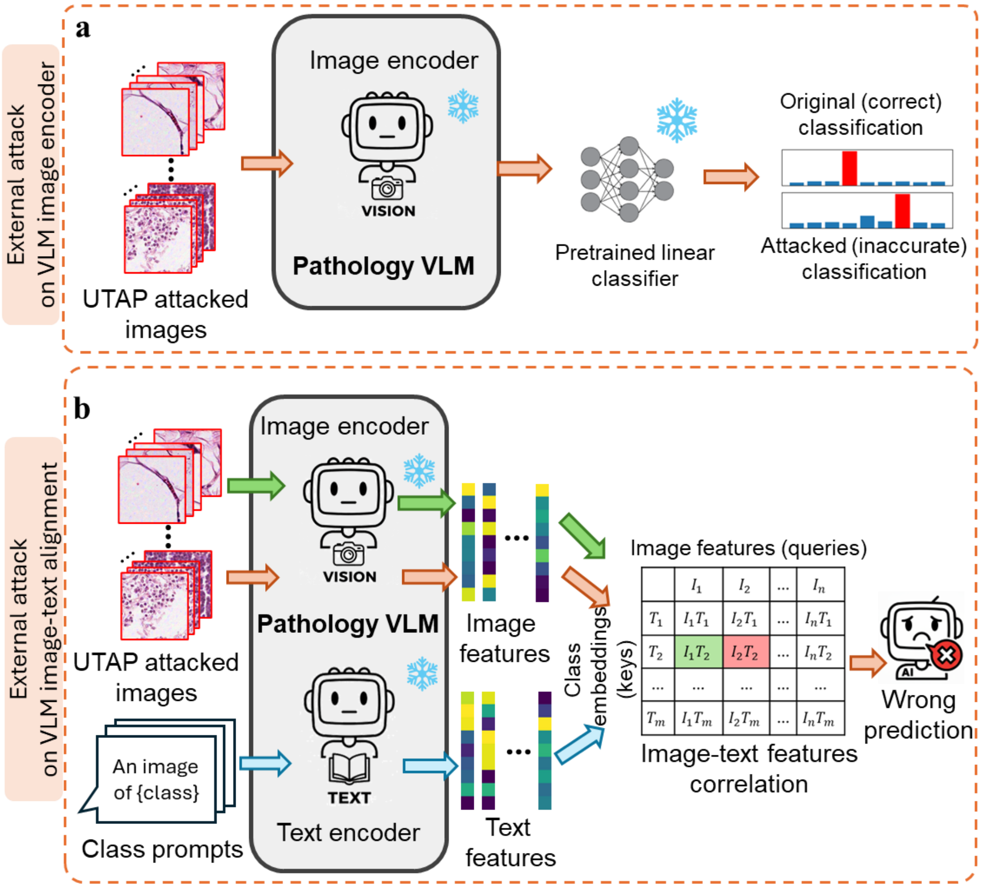

Supplementary Fig. S8. Transferability of universal adversarial perturbations to patch-level pathology vision language models (VLMs). (a) Schematic of the external attack targeting the VLM's frozen image encoder, evaluated via a pre-trained linear classifier. The application of the fixed UTAP microscopic perturbation results in a substantial reduction of the classification accuracy from 95.6% to 78.9%. (b) Workflow of the external attack on VLM image-text alignment, illustrating the disruption of the multimodal representation space when correlating perturbed image features with text features extracted from class-specific prompts (e.g., "An image of {class}"). Under this visual domain attack, the zero-shot image-text alignment accuracy is degraded from 79.1% to 69.8%.

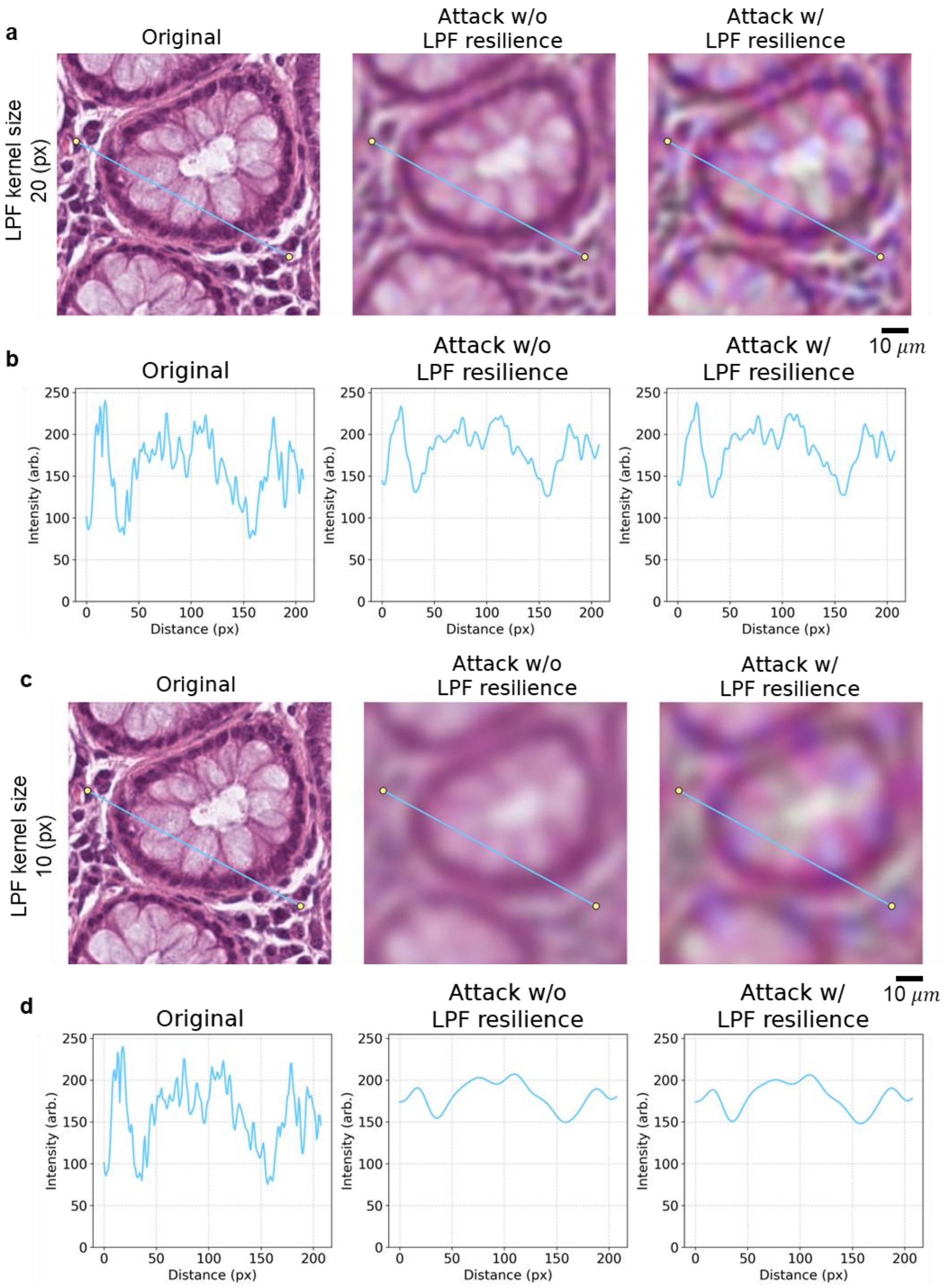

Supplementary Fig. S9. Evaluation of Low-Pass Filter (LPF) defense and adaptive adversarial resilience at reduced kernel sizes. Visual and physical impact of the LPF application on microscopic images using 20 (a,b) and 10 (c,d) pixel kernel sizes. The panels compare the original tissue, the standard attack without LPF resilience, and the adaptive attack with LPF resilience, alongside their corresponding 1D spatial intensity profiles.

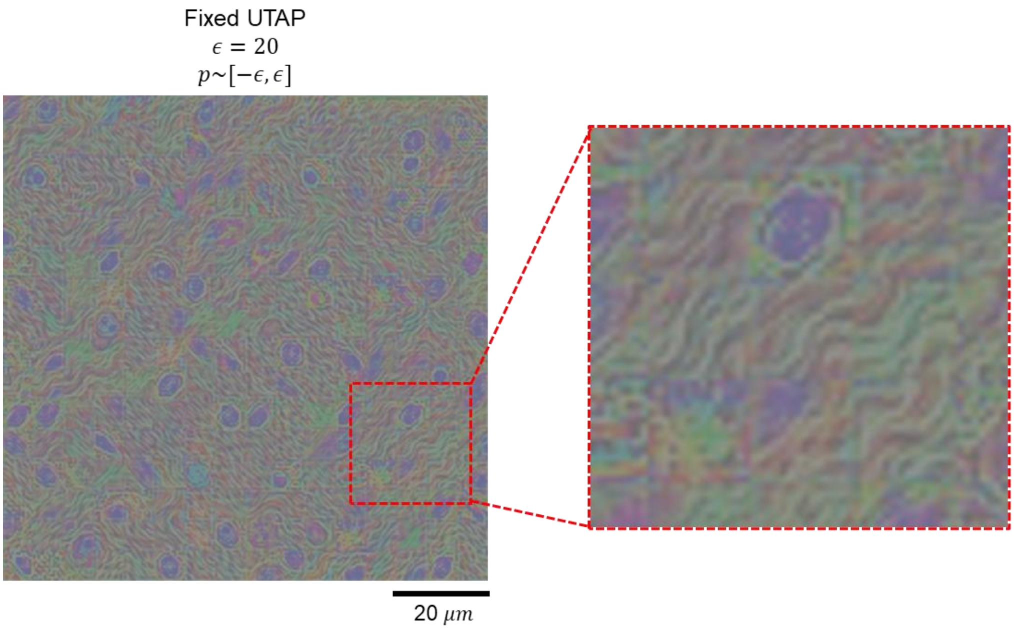

Supplementary Fig. S10. High-magnification visualization of the optimized universal and transferable adversarial perturbation. The zoomed-in inset highlights the distinct grid-like artifacts and periodic spatial textures inherent to the generated perturbation pattern.

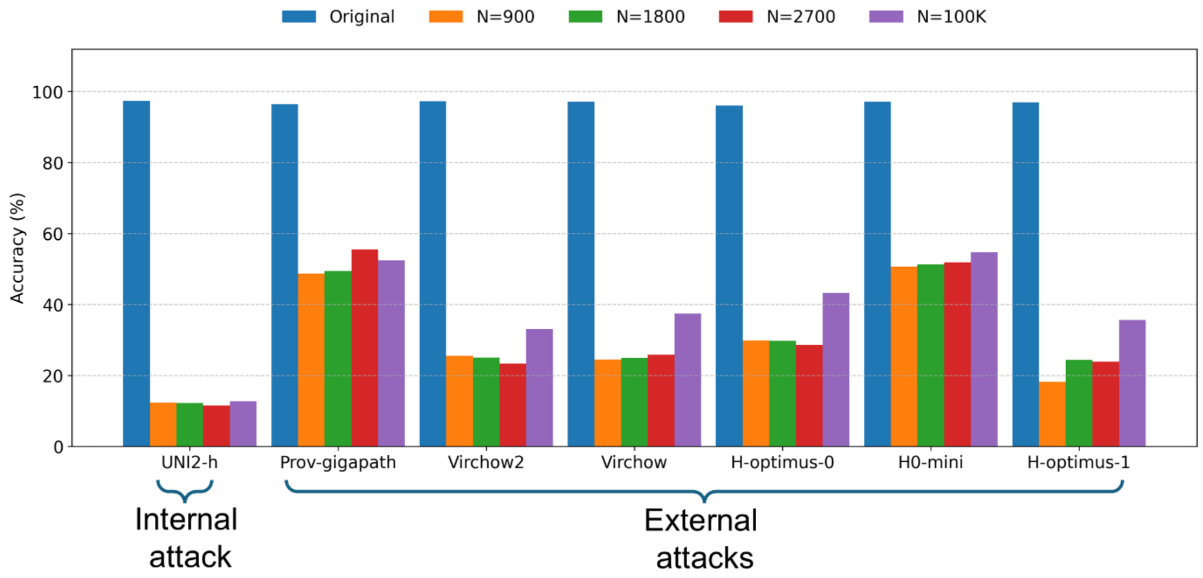

Supplementary Fig. S11. Classification accuracy of the microscopic images attacked by UTAP optimized with different number of training images (N). The purple bars indicate using the full training dataset, where $N = 100K$.

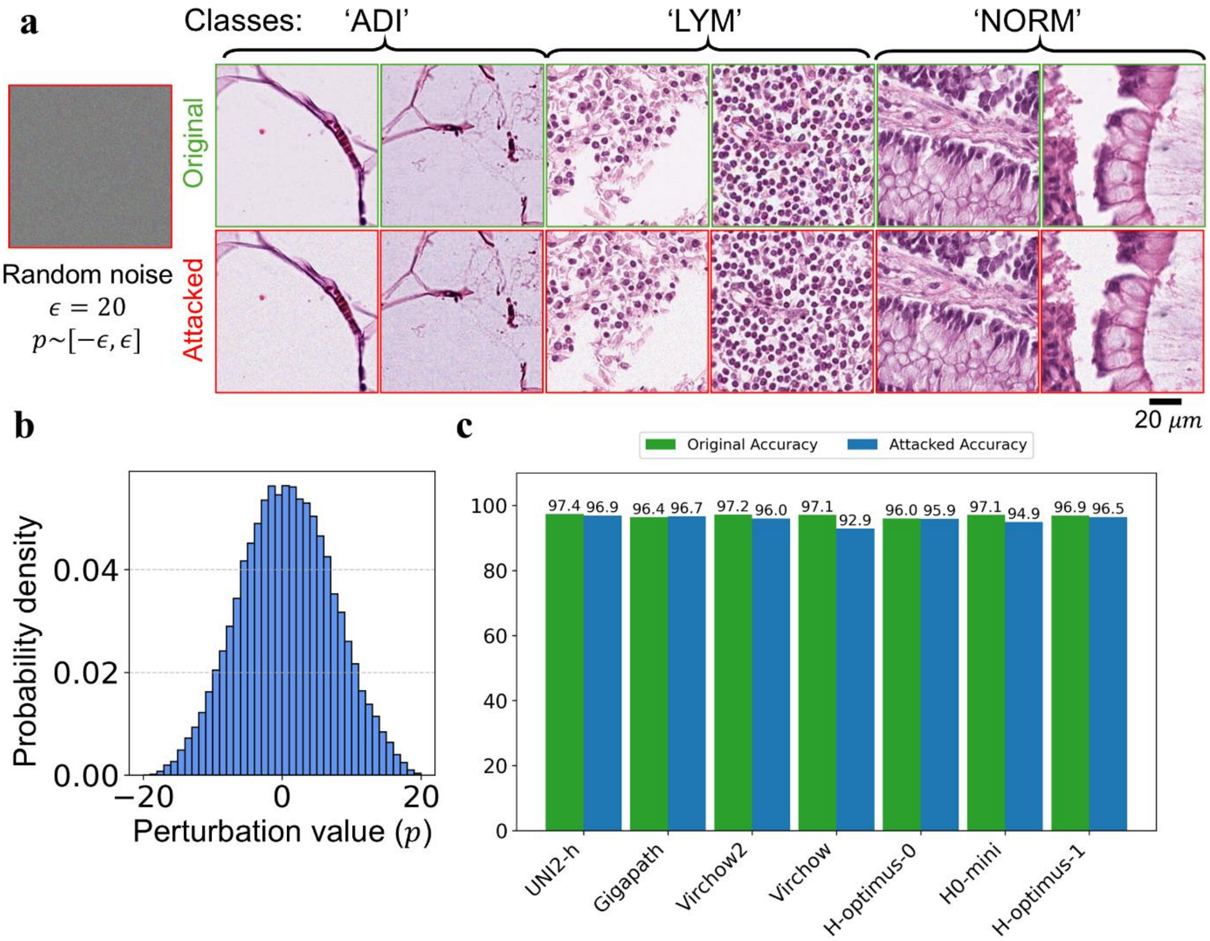

Supplementary Fig. S12. Random noise perturbation attack results on CRC-100K dataset[1]. (a) Visualization of random noise with the original and the attacked microscopic images of three classes sampled from the CRC-100K dataset. (b) The histogram of random noise averaged across the RGB color channels, bounded in the range of $\pm\epsilon$. (c) The original (green) and the attacked (blue) classification accuracies across all foundation models. Note that we sampled the random perturbation uniformly across the RGB channels, but here show it by averaging across channels, which follows an Irwin-Hall distribution[10].

References

1. Kather, J. N., Halama, N. & Marx, A. 100,000 histological images of human colorectal cancer and healthy tissue. https://zenodo.org/records/1214456 https://doi.org/10.5281/zenodo.1214456 (2018).
2. Chen, R. J. *et al.* Towards a general-purpose foundation model for computational pathology. *Nature Medicine* **30**, 850–862 (2024).
3. Xu, H. *et al.* A whole-slide foundation model for digital pathology from real-world data. *Nature* **630**, 181–188 (2024).
4. Zimmermann, E. *et al.* Virchow2: Scaling Self-Supervised Mixed Magnification Models in Pathology. (2024).
5. Daisuke, K. & Shumpei, I. Histology images from uniform tumor regions in TCGA Whole Slide Images. https://zenodo.org/records/3373439 https://doi.org/10.5281/zenodo.3373439 (2020).
6. Polónia, A., Eloy, C. & Aguiar, P. BACH Dataset : Grand Challenge on Breast Cancer Histology images. https://zenodo.org/records/3632035 (2019).
7. Spanhol, F. A., Oliveira, L. S., Petitjean, C. & Heutte, L. A Dataset for Breast Cancer Histopathological Image Classification. *IEEE Transactions on Biomedical Engineering* **63**, (2016).
8. Lou, S. *et al.* A large histological images dataset of gastric cancer with tumour microenvironment annotation for AI. *Scientific Data* **12**, 138 (2025).
9. Borkowski, A. A. *et al.* Lung and Colon Cancer Histopathological Image Dataset (LC25000). (2019).
10. PHILIP, H. The Distribution of Means for Samples of Size N Drawn from a Population in Which the Variate Takes Values Between 0 and 1, All Such Values Being Equally Probable. *Biometrika* **19**, 240–244 (1927).
